# The Transcriptomic Signature Of Disease Development And Progression Of Nonalcoholic Fatty Liver Disease

**DOI:** 10.1038/s41598-017-17370-6

**Published:** 2017-12-08

**Authors:** Sophie Cazanave, Alexei Podtelezhnikov, Kristian Jensen, Mulugeta Seneshaw, Divya P. Kumar, Hae-Ki Min, Prasanna K. Santhekadur, Bubu Banini, Adolfo Gabriele Mauro, Abdul M. Oseini, Robert Vincent, Keith Q. Tanis, Andrea L. Webber, Liangsu Wang, Pierre Bedossa, Faridoddin Mirshahi, Arun J. Sanyal

**Affiliations:** 1 Division of Gastroenterology, Hepatology and Nutrition, Department of Internal Medicine, Virginia Commonwealth University, Richmond, VA USA; 2 Merck Research Laboratories, Kenilworth, NJ USA; 3 VCU Pauley Heart Center, Virginia Commonwealth University, Richmond, VA USA; 4 Department of Pathology, Hospital Beaujon, University Paris-Diderot, Paris, France

## Abstract

A longitudinal molecular model of the development and progression of nonalcoholic fatty liver disease (NAFLD) over time is lacking. We have recently validated a high fat/sugar water-induced animal (an isogenic strain of C57BL/6 J:129S1/SvImJ mice) model of NAFLD that closely mimics most aspects of human disease. The hepatic transcriptome of such mice with fatty liver (8 weeks), steatohepatitis with early fibrosis (16–24 weeks) and advanced fibrosis (52 weeks) after initiation of the diet was evaluated and compared to mice on chow diet. Fatty liver development was associated with transcriptional activation of lipogenesis, FXR-RXR, PPAR-α mediated lipid oxidation and oxidative stress pathways. With progression to steatohepatitis, metabolic pathway activation persisted with additional activation of IL-1/inhibition of RXR, granulocyte diapedesis/adhesion, Fc macrophage activation, prothrombin activation and hepatic stellate cell activation. Progression to advanced fibrosis was associated with dampening of metabolic, oxidative stress and cell stress related pathway activation but with further Fc macrophage activation, cell death and turnover and activation of cancer-related networks. The molecular progression of NAFLD involves a metabolic perturbation which triggers subsequent cell stress and inflammation driving cell death and turnover. Over time, inflammation and fibrogenic pathways become dominant while in advanced disease an inflammatory-oncogenic profile dominates.

## Introduction

Nonalcoholic fatty liver disease is the most common cause of chronic liver disease in the Western world and is rapidly increasing in prevalence globally^1^. It may manifest itself as nonalcoholic fatty liver (NAFL) or nonalcoholic steatohepatitis (NASH). While it was generally assumed that only NASH progressed to cirrhosis and end-stage liver disease, recent studies indicate that NAFL can also lead to progressive fibrosis^2–4^.

A vast amount of literature has already accumulated documenting activation of specific cellular pathways in the genesis and evolution of NASH^5,6^. It is however not known if these pathways all get turned on and remain turned on throughout the course of the disease. The relevance of when specific pathways are activated during disease evolution to cirrhosis is also unknown. Current drug development is based on the assumption that the molecular target for a given drug is equally relevant for all patients with NASH; however the failure to achieve a therapeutic response in only 40–50% of treated patients despite similar drug compliance indicates that the population is heterogeneous with respect to treatment response. A potential explanation for this could be that different molecular pathways are differentially activated at various points in disease progression. While this is far from proven, longitudinal assessment of the transcriptome with evolution of the disease is a required first step to address this possibility and inform future Precision Medicine approaches for NASH. Indeed, such transcriptomic models have provided the foundational basis for Precision Medicine approaches for many cancers^7^.

The ideal method to develop a dynamic molecular model of NASH development and progression is to obtain transcriptomic data and relate it to liver histology at multiple time points over a course of many years. Unfortunately, there is still a great paucity of such data and current attempts to evaluate the NASH transcriptome are entirely cross-sectional^8–12^. Such studies however do not account for changes with time and disease evolution and only provide indirect evidence about the temporal sequence of changes and their relationship to disease progression. A potential alternative approach could be to obtain longitudinal data in an animal model of NASH that has been validated to closely reflect human disease.

Recently, we have described a diet-induced animal model of NAFLD (DIAMOND) which faithfully develops steatosis followed by steatohepatitis and then progressive fibrosis and even HCC following initiation of a high fat diet with ad libitum administration of a glucose-fructose containing water^13^. This model matches human disease with respect to lack of specific gene knockouts, induction by relevant diet, development of insulin resistance and obesity, histological phenotype including classical ballooning and Mallory Denk body formation and activation of molecular pathways known to be relevant for human disease. Importantly, by gene-set enrichment analysis, there is a concordance with human data sets from cross sectional studies of NAFLD and NASH related cirrhosis.

In this study, the initial transcriptomic readouts from DIAMOND mice were expanded to include early time points where NAFL was present, intermediate time points where steatohepatitis with none or early fibrosis was present and late time points where advanced fibrosis with or without HCC was present. These were used to develop a dynamic molecular model of NAFLD development and progression in this mouse model of NAFLD. The concordance of these data to human NAFLD was confirmed for selected targets known to be relevant for human disease.

## Results

A total of 4–5 DIAMOND mice each were evaluated 8 weeks, 24 weeks and 52 weeks following initiation of WD SW diet. At each time point, 4-5 mice who received chow diet were evaluated and served as controls. As expected^13^, a WD SW diet led to weight gain, insulin resistance, and dyslipidemia (Supplementary Table S1). At 8 weeks after initiation of the WD SW diet, all mice had a fatty liver and only one mouse had mild cytological ballooning (Supplementary Fig. S1). At this time point, none of the mice studied displayed any portal inflammation or perisinusoidal or portal fibrosis. By 16-24 weeks, more than half of the mice had steatohepatitis with at least stage 1 fibrosis^13^. At 24 weeks, the mean inflammation grade and hepatocellular ballooning grade were 1 ± 0.0 and 0.5 ± 0.29 respectively and significantly different from that seen at the 8-week time point when NAFL was present. By week 52, there was florid severe steatohepatitis and all the mice had stage 2–3 fibrosis (Stage 2 fibrosis in 3/5 mice and stage 3 fibrosis in 2/5). The NAFLD activity score (NAS) was 3.8 ± 0.2 at 8 weeks and was driven mainly by steatosis. At weeks 24, the NAS ranged from 3 to 5 (4.2 ± 0.5) and was driven by steatosis, inflammation and also ballooning. By week 52, the steatosis scores had decreased but the NAS remained high due to active inflammation and ballooning.

### Changes in disease-related pathways with disease development and evolution

#### Changes in Lipid Metabolic pathways

**Figure 1:**
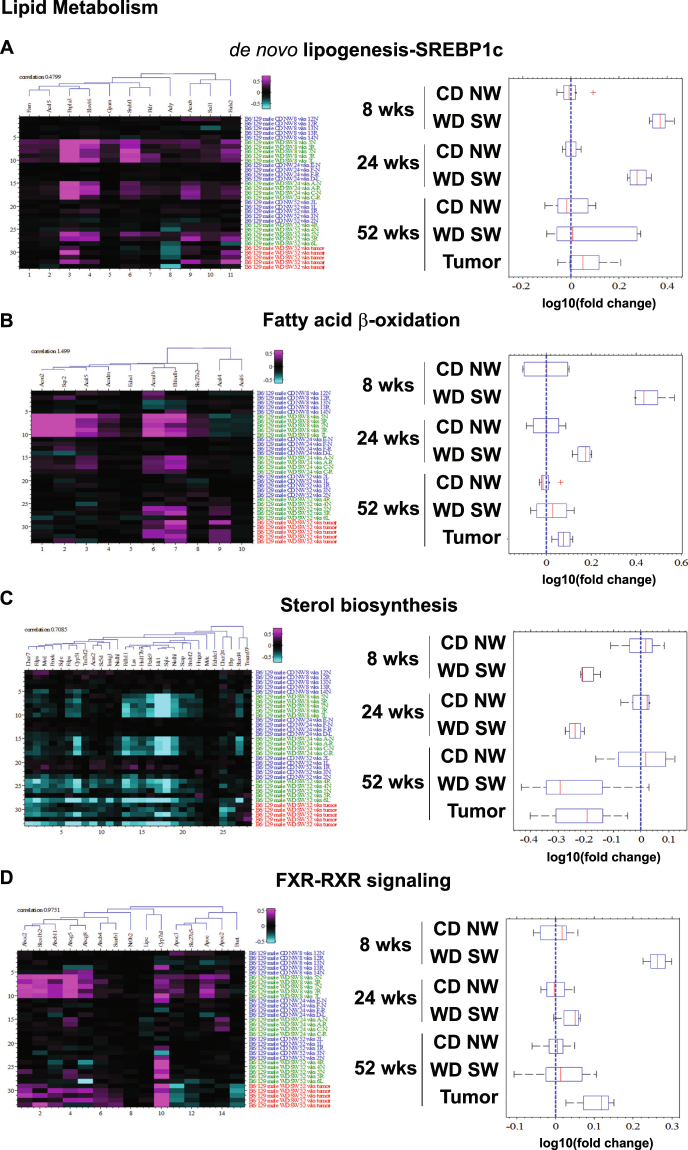
Changes in metabolic pathways. Heat maps resulting from hierarchical clustering and box plots with averages for genes implicated in (**A**) *de novo* lipogenesis (11 genes averaged), (**B**) Fatty acid β-oxidation (8 genes averaged), (**C**) Sterol biosynthesis (22 genes averaged) or (**D**) FXR-RXR signaling (14 genes averaged) pathways with a fold-change greater than ±1.5 from WD SW liver samples as compared to CD NW at 8, 24 and 52 weeks or from liver tumors at 52 weeks as compared to WD SW 52 weeks with a false discovery rate (FDR) <0.1. Boxes show 25^th^ and 75^th^ percentile, whiskers show 5^th^ and 95^th^ percentile, red line is the median average score, and blue dash line shows no change compared with CD NW at 8, 24 and 52 weeks or compared to WD SW 52 weeks for liver tumors.

There was a substantial perturbation in the level of expression of genes involved in lipid metabolism with the development of NAFL eight weeks after initiation of a WD SW diet with activation of both *de novo* lipogenesis-associated genes and those associated with lipid oxidation (Fig. 1, Supplementary Tables S2–S6). At the level of individual genes, the key genes of fatty acid oxidation such as *acetyl-CoA C-acyltransferase 2* (*Acaa2*), *acetyl-CoA C-acyltransferase 1b* (*Acaa1b*) and *enoyl-CoA hydratase and 3-hydroxyacyl CoA dehydrogenase* (*Ehhadh*) or *long chain fatty acid CoA ligase 5* (*Acsl5*) and *elongation of very long chain fatty acids* (*Elovl6*) were upregulated in response to WD SW-feeding (Fig. 1A and B). In correlation with the major changes in pathways related to lipid metabolism, the top up-regulated genes at 8 weeks included a major carrier of *lipid apolipoprotein A4* (*ApoA4*) and the triacylglycerol lipase *adiponutrin/Patatin-like phospholipase domain-containing protein 3* (*Pnpla3*) (Supplementary Table S5). Also, IPA identified *peroxisome proliferator-activated receptor* α (*Pparα* which is involved in the metabolic control of the expression of genes encoding for fatty acid oxidation enzymes) as a top upstream regulator being activated and the *peroxisomal acyl-coenzyme A oxidase 1* (*Acox1* which regulates fatty acid oxidation) as a top upstream regulator being inhibited (Supplementary Table S3).

In contrast to humans, there was however a downregulation of HMG CoA reductase the rate limiting enzyme for cholesterol synthesis (Fig. 1C). This was accompanied by suppression of several additional genes involved in cholesterol synthesis such as farnesyl diphosphate synthetase, 3 *β-hydroxysteroid dehydrogenase type 4* (*Hsd3b4*), a key enzyme in the biosynthesis of all classes of hormonal steroids and squalene epoxidase (Supplementary Table S5).

The nuclear receptor liver X receptor (LXR) and farnesoid X receptor (FXR) are both essential regulators of cholesterol homeostasis^14^. Upregulation of the enzyme *cholesterol 7 alpha-hydroxylase* (*Cyp7a1*) (Fig. 1D) was indicative of LXR activation. This was further confirmed by quantitative real time PCR (Supplementary Fig. S2). FXR activation normally suppresses Cyp7a1 and activation of Cyp7a1 indicated a tilt in favor of LXR rather than FXR activation. However, several other FXR targets were upregulated indicating dysregulation of the LXR-FXR metabolic axis. Examples of activated FXR targets included the ATP binding cassette bile salt export pump (*abc11*) and the canalicular multispecific organic anion transporter (*abcc*2) and canalicular cholesterol transporters *abcg5* and *abcg8* **(**Fig. 1D **)**.

Genes implicated in phospholipids biosynthesis (*acylglycerol-*3*-acyltransferase*, *phosphatidic acid phosphatase* or *CDP-diacylglycerol synthase*) were not significantly changed (Supplementary Excel Sheet S1–S4). However, the expression of the rate-limiting enzyme phosphatidylethanolamine methyltransferase (*pemt*), which converts phosphatidylethanolamine (PE) to phosphatidylcholine (PC) in the liver was significantly decreased at 24 and 52 weeks (Supplementary Excel Sheet S1–S4 **)**.

**Figure 2:**
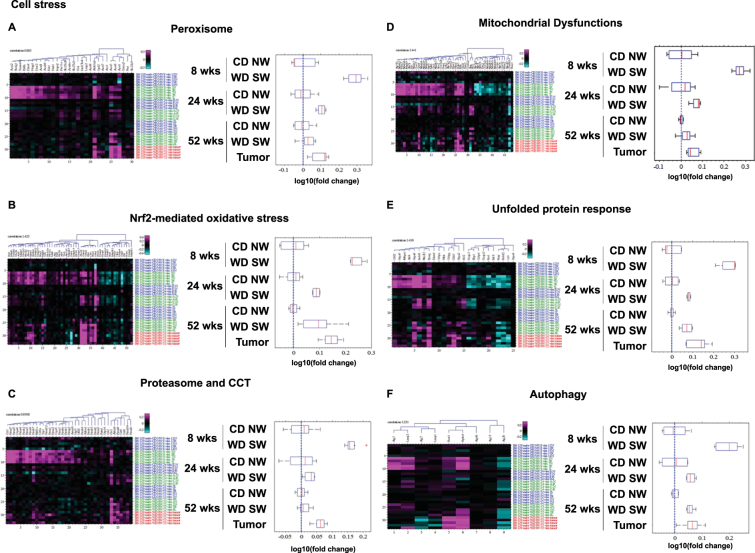
Changes in cell stress-related pathways. Heat maps resulting from hierarchical clustering and box plots with averages for genes implicated in (**A**) Peroxisome (30 genes averaged), (**B**) Nrf2-mediated oxidative stress (37 genes averaged), (**C**) Proteasome (48 genes averaged), (**D**) Mitochondrial dysfunctions (28 genes averaged), (**E**) Unfolded protein response (15 genes averaged) and (**F**) Autophagy (6 genes averaged) signaling pathways with a fold-change greater than ±1.5 from WD SW liver samples as compared to CD NW at 8, 24 and 52 weeks or from liver tumors at 52 weeks as compared to WD SW 52 weeks with a false discovery rate (FDR) <0.1. Boxes show 25^th^ and 75^th^ percentile, whiskers show 5^th^ and 95^th^ percentile, red line is the median average score, and blue dash line shows no change compared with CD NW at 8, 24 and 52 weeks or compared to WD SW 52 weeks for liver tumors.

With the development of steatohepatitis (weeks 16–24)^13^, some of the early lipid metabolic pathway changes persisted (Supplementary Table S4). SREBP1c-mediated *de novo* lipogenesis pathway (Fig. 1A) remained increased with *ApoA4*, *lipoprotein lipase precursor* (*Lpl*) and *Pnpla3* as top down-regulated genes (Supplementary Table S5), and *Pparα* as a top upstream activated regulator and *Acox1* as a top upstream inhibited regulator (Supplementary Table S3). Also, there was a significant increase in protein expression of fatty acid synthetase (FAS) as well as acetyl CoA carboxylase (ACC) (Supplementary Fig. S3). However, β-oxidation/fatty acid synthesis (Fig. 1B), FXR/RXR activation (Fig. 1D), and mitochondrial/peroxisomal fatty acid oxidation (Fig. 2A) pathways were maximally altered at 8 weeks in the WD SW mice but the degree of genes expression change in these pathways was decreased at 24 weeks as compared to 8 weeks. Sterol biosynthesis (Fig. 1C) remained decreased, with *Hsd3b4*, *Sqle*, and the *25-hydroxycholesterol* 7*-alpha-hydroxylase Cyp7b1* (which catalyzes the first reaction in the cholesterol catabolic pathway) as top down-regulated genes (Supplementary Table S6). Additionally, downregulation of *3 hydroxysterol Δ14-reductase Tm7sf2* (an endoplasmic reticulum enzyme involved with cholesterol biosynthesis) and *insulin induced gene 1 Insig1* (whose overexpression attenuates hepatic steatosis and plasma cholesterol levels induced by an atherogenic diet^15^) was noted (Fig. 1C). Finally, gene expression for the cholesterogenic cytochrome P450 *lanosterol 14α-Demethylase* (*Cyp8 wks51*) was also decreased (Fig. 1C), an enzyme that catalyzes demethylation of lanosterol in the cholesterol synthesis pathway using cytochrome P450 reductase (POR) as an obligatory redox partner; and the *POR* gene was identified by IPA as a major top upstream regulator (Supplementary Table S3).

By week 52, when bridging fibrosis had developed, pathways related to lipid metabolism, sterol biosynthesis were less activated compared to earlier time points (Fig. 1). Also, *glycerol-3-phosphate dehydrogenase 1* (*Gpd1*, required for triglyceride synthesis) was identified by IPA as a top upstream regulator (Supplementary Table S3). FAS and ACC protein expression remained increased in the WD SW-fed mice (Supplementary Fig. S3). Interestingly, IPA analysis inferred that microRNA miR-34a could represent a top upstream regulator in WD SW-fed mice at 52 weeks (Supplementary Table S3). Also, *insulin like growth factor binding protein 2* (*Igfbp2*), was among the top down-regulated genes (Supplementary Table S6), a result consistent with previous studies indicating that Igfbp2 modulates insulin sensitivity and that low levels of Igfbp2 are associated with type 2 Diabetes (T2DM)^16^. In correlation with this observation, WD SW-fed mice display marked insulin-resistance at 52 weeks^13^. Taken together, these results indicate that genes related to lipid metabolic pathways are activated early in the course of NAFLD (simple steatosis at 8 weeks) and such changes dampen with further progression to steatohepatitis and increasing fibrosis.

#### Cell stress-related pathway activation

**Figure 3:**
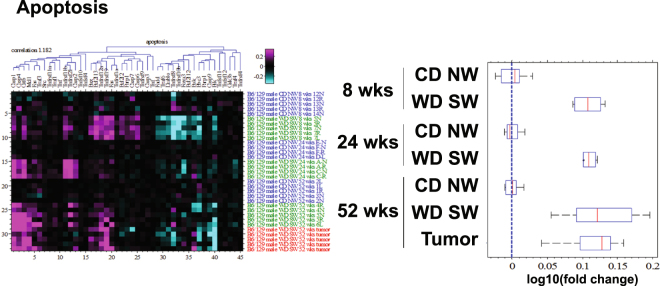
Changes in apoptosis pathways. Heat maps resulting from hierarchical clustering and box plots with averages for genes implicated in apoptosis pathways (25 genes averaged) with a fold-change greater than ±1.5 from WD SW liver samples as compared to CD NW at 8, 24 and 52 weeks or from liver tumors at 52 weeks as compared to WD SW 52 weeks with a false discovery rate (FDR) <0.1. Boxes show 25^th^ and 75^th^ percentile, whiskers show 5^th^ and 95^th^ percentile, red line is the median average score, and blue dash line shows no change compared with CD NW at 8, 24 and 52 weeks or compared to WD SW 52 weeks for liver tumors.

Oxidative stress, unfolded protein response and autophagy are well established cell stress pathways in NASH in humans^17^. Cell stress was also apparent at 8 weeks as evidenced by the activation of pathways related to Nrf2-mediated oxidative stress (Fig. 2B), proteasome activity (Fig. 2C), mitochondrial dysfunction (Fig. 2D), unfolded protein response (UPR) (Fig. 2E), autophagy (Fig. 2F) and apoptosis (Fig. 3). In accord with a cellular stress response, IPA identified autophagy-related gene *Rictor*, Nrf2 (*Nfe2l2*) and p53 (*Tp53*) as top upstream regulators (Supplementary Table S3).

The top up-regulated genes included the *carbonic anhydrase 3* (*CA3*), an enzyme with cytoprotective action against oxidative stress and *haptoglobin* (*Hh*), an acute phase protein with antioxidant properties (Supplementary Table S5). Other hepatic genes related to oxidative stress responses such as genes of the glutathione S-transferase family (*Gsta1*, *Gstm2*, *Gstm3* and *mGst3*) or Nrf2-regulated *NAD(P)H quinone dehydrogenase 2* (*Nqo2*) and *glutamate-cysteine ligase catalytic subunit* (*Gclc*) were upregulated in WD SW-fed mice; while *Kelch-like ECH-associated protein 1* (*Keap-1*), a negative regulator of Nrf2 expression was downregulated (Fig. 2B).

Similarly, genes coding for proteasome subunits (*Psma4*, *Psma7*, *Psmb2*, *Psmb3*, *Psmb6*, *Psmb7*, *Psmc1*, *Psmc2*, *Psmc3*, *Psmc4*, *Psmc5*, *Psmd7*, *Psmd12*) (Fig. 2C), for the mitochondrial *voltage-dependent anion channel 3* (*Vdac3*) (Fig. 2D), for the endoplasmic reticulum (ER) stress-induced transcription factor CCAAT/enhancer binding protein CHOP (*ddit3*) and the activating transcription factor 4 (*Atf4*) (Fig. 2E and Supplementary Fig. S2) were all upregulated.

The gene expression of several autophagy-related proteins such as the *autophagy related 3* (*Atg3*), *lysosome 2* (*Lamp2*) and *beclin 1* (*Becn1*) were all upregulated in DIAMOND mice at 8 weeks (Fig. 2F). Expression of several genes implicated in apoptosis was also moderately upregulated such as the death receptor *Fas*, the executioner *caspases 6 and 7* (*Casp6, Casp7*), the enzyme *poly ADP ribose polymerase* (*Parp1*) and the Bcl2 protein members *Bcl-w* (*Bcl2l2*) and Bax (Fig. 3).

Interestingly, by the time steatohepatitis developed (measured at week 24), cell stress-related pathway activation was dampened compared to both chow-fed controls and WD SW-fed mice at 8 weeks when only NAFL with some minor inflammation was present. Specifically, Nrf2-dependent oxidative stress, proteasome activity, mitochondrial dysfunction, UPR, and autophagy (Fig. 2B–F) all decreased but the increase in apoptotic pathway persisted (Fig. 3). This decrease at week 24 was however transitory and probably artefactual given that the expression of these genes were increased at week 52 when aggressive NASH and bridging fibrosis were present. Further, PCR and Western blot data confirmed continued activation of these pathways throughout the course of steatohepatitis including week 24 (Supplementary Fig. S2 and S3). These results indicate that following cell injury-related pathways are activated progressively at both early and late time points.

#### Increasing inflammatory signaling is a hallmark feature of progressive NASH

Activation of gene expression for the inflammatory mitogen-activated protein kinases *Jnk2* (*Mapk9*), *p38α* (*Mapk14*) and *Erk2* (*Mapk1*) was noted with development of NAFL (week 8) even in the absence of histologically evident inflammation (Supplementary Fig. S2). These further increased with development of steatohepatitis (week 24). By 24 weeks, phosphorylated and activated JNK and p44/42 were maximally increased (Supplementary Fig. S3)

**Figure 4:**
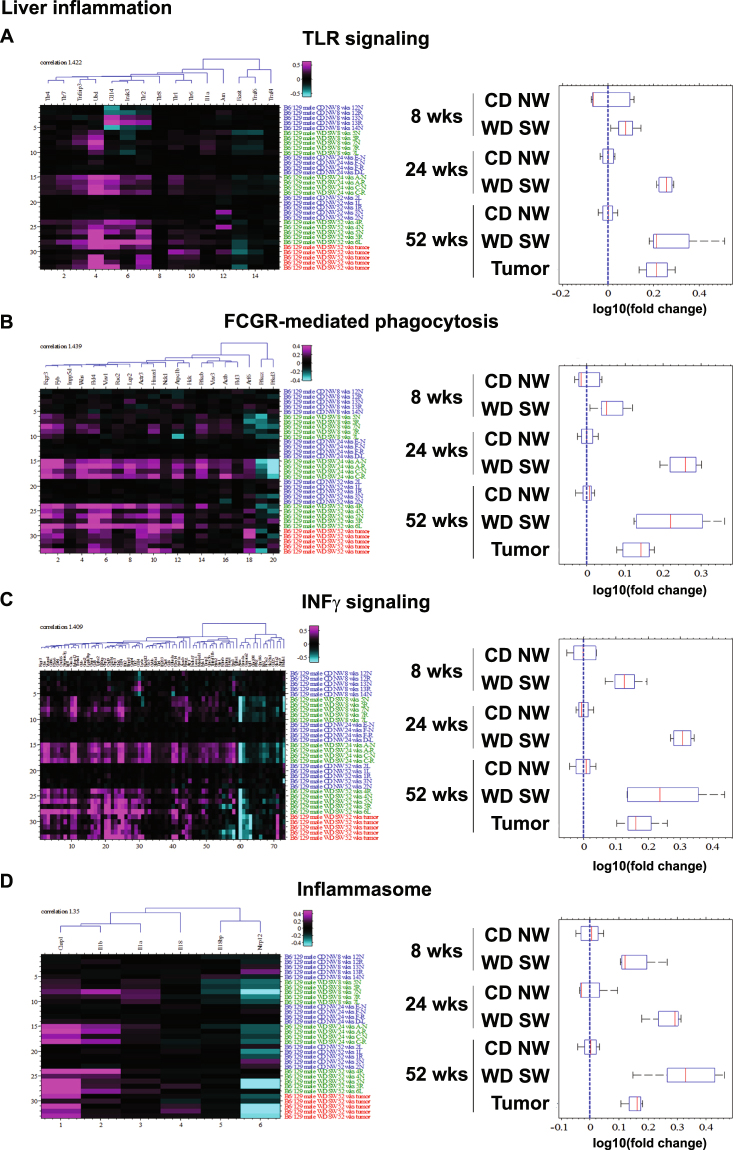
Changes in inflammatory pathways. Heat maps resulting from hierarchical clustering and box plots with averages for genes implicated in (**A**) TLR signaling (12 genes averaged), (**B**) Fcgr-mediated phagocytosis (18 genes averaged), (**C**) INFγ signaling (58 genes averaged), (**D**) Inflammasome (4 genes averaged) pathways with a fold-change greater than ±1.5 from WD SW liver samples as compared to CD NW at 8, 24 and 52 weeks or from liver tumors at 52 weeks as compared to WD SW 52 weeks with a false discovery rate (FDR) <0.1. Boxes show 25^th^ and 75^th^ percentile, whiskers show 5^th^ and 95^th^ percentile, red line is the median average score, and blue dash line shows no change compared with CD NW at 8, 24 and 52 weeks or compared to WD SW 52 weeks for liver tumors.

The canonical pathways for Toll-like receptor (TLR) signaling (Fig. 4A), Fcgr-mediated phagocytosis (Fig. 4B), interferon-γ (INFγ)-signaling (Fig. 4C) and inflammasome (Fig. 4D) were all significantly increased at 24 weeks. Genes encoding for *toll-like receptor 2* (*Tlr2*) (Fig. 4A), for Fc receptor *Fcgr3* (Fig. 4B), for chemokines (*Ccl3*, *Ccl4*, *Ccl5*, *Ccl6*, *Cxcl9*, *Cxcl14*, *Cxcl16*) (Supplementary Fig. S2 and S4), for inflammation-induced transcription factors *Jun* and *Fos* (Supplementary Fig. S5), for hedgehog-activated transcription factor *Gli3* (Supplementary Fig. S6), for the inflammatory *caspase 1* (*Casp1*) and the pro-inflammatory cytokine *Il1b* (Fig. 4D) were all upregulated in WD SW-fed mice. Together with *IL1-b* and *caspase-1*, *apoptosis-associated speck-like protein containing a carboxy-terminal CARD* (*ASC*, a bipartite adapter protein that regulates inflammasome formation) mRNA levels was increased (Supplementary Fig. S2). Activation of the inflammasome was further confirmed by immunohistochemical staining for ASC protein in both mice and humans (Supplementary Fig. S7). Finally, IPA also identified the cytokine INFγ as a top upstream regulator being activated (Supplementary Table S2).

The hepatic inflammatory response in humans as well as the DIAMOND mice often consists of a mixed inflammatory cell infiltrate^18^. Both *eosinophil-associated ribonuclease A* (*Ear*) *2* and *3* were included in the top up-regulated genes at 24 and 52 weeks (Supplementary Table S5); and mouse Ear2 is a chemoattractant for dendritic cells^19^, a population of specialized hematopoietic cells that may contribute to inflammation and fibrosis progression in NAFLD^20^.

With continued disease progression (week 52) and despite similar histological activity related to inflammation compared to earlier time points, the transcriptomic signature of WD SW-fed mice was dominated by pro-inflammatory pathways and the 5 top predominant canonical pathways included Fc receptor-mediated phagocytosis in macrophages and monocytes (p = 4.3E-06, ratio 11/87), histidine degradation III (p = 3.8E-04, ratio 3/7), actin nucleation by actin-rich structure (ARS)-WASp complex (p = 1.0E-03, ratio 6/51), epithelial adherens junction signaling (p = 1.04E-03, ratio 10/135) and production of nitric oxide and reactive oxygen species in macrophages (p = 1.47E-03, ratio 11/166) (Supplementary Table S2). Genes coding for the Fc portion of immunoglobulin G *Fcgr3a/Fcgr3b* expressed by NK cells, *ear2*, the *complement subcomponent C1q chain b* (*C1qb*), *cd52* (a protein present on the surface of mature lymphocytes, monocytes and dendritic cells) and *macrophage-expressed gene 1* (*Mpeg1*) were all included in the top upregulated genes (Supplementary Table S3). INFγ was again identified as a top upstream regulator being activated at this time point (Supplementary Table S2), and genes encoding for chemokines (*Ccl4*, *Ccl5*, *Cxcl9*) (Supplementary Fig. S6), *Casp1* and *Il1b* (Fig. 4D) were still upregulated. These results indicate a progressive activation of inflammatory pathways simultaneously to the induction of cell injury-related pathways.

#### Fibrogenic signaling peaks before histologically maximal fibrosis is seen

**Figure 5:**
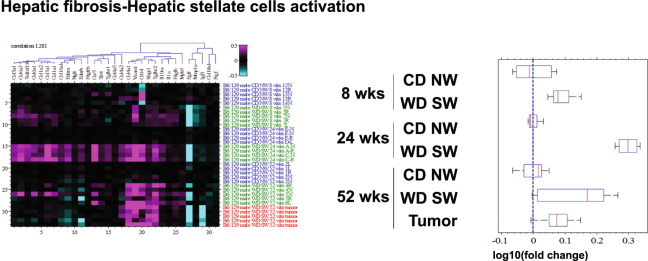
Changes in fibrosis pathways. Heat maps resulting from hierarchical clustering and box plots with averages for genes implicated in fibrosis pathways (26 genes averaged) with a fold-change greater than ±1.5 from WD SW liver samples as compared to CD NW at 8, 24 and 52 weeks or from liver tumors at 52 weeks as compared to WD SW 52 weeks with a false discovery rate (FDR) <0.1. Boxes show 25^th^ and 75^th^ percentile, whiskers show 5^th^ and 95^th^ percentile, red line is the median average score, and blue dash line shows no change compared with CD NW at 8, 24 and 52 weeks or compared to WD SW 52 weeks for liver tumors.

Modest activation of fibrogenic pathways was observed even when there was no discernable fibrosis on histological assessment (Fig. 5); the glycoprotein *alpha-1-anti-trypsine* (*Serpina1*), for which deficiency is linked to both cirrhosis and primary liver cancer was one of the top most down-regulated gene (Supplementary Table S6) and the alcohol metabolizing enzyme *alcohol dehydrogenase 1 C* (*Adh1C*), which activity relates to alcoholic liver cirrhosis risk^21^, was upregulated (Supplementary Table S5).

With the development of steatohepatitis and stage 1-2 fibrosis by week 16–24^13^, the canonical pathway for hepatic fibrosis/hepatic stellate cells activation was markedly increased at week 24 (Fig. 5), consistent with an increased in α-smooth muscle actin (α-SMA) and desmin levels observed in these mice at that time point^13^. The top up-regulated genes included the macrophage-derived metalloelastase (*Mmp12*) (Supplementary Table S5), a metalloproteinase which degrades extracellular matrix components as part of the tissue remodeling/wound repair process occurring during progressive liver fibrosis^22^. Genes coding for several collagen type (*Col1a1*, *Col1a2*, *Col3a1*, *Col4a2*, *Col5a1*, *Col6a1*, *Col6a3*, *Col14a1*), for *TIMP metalloproteinase inhibitor 1 and 3* (*Timp1*, *Timp3*), for *Mmp13* and for *vascular cell adhesion protein 1* (*Vcam1*) were also all upregulated in WD SW-fed mice (Fig. 5 and Supplementary Fig. S2), attesting to hepatic stellate cells activation, a central event in hepatic fibrosis.

Interestingly, with development of bridging fibrosis at week 52, changes in pathways related to hepatic fibrosis/hepatic stellate cells activation decreased as compared to 24 weeks and were mainly limited to increase in genes for *Col4a1*, *Mmp12*, *Mmp13* and *Vcam1* (Fig. 5). Taken together the results indicate an upregulation in genes related to fibrosis is seen predominantly at early stage of steatohepatitis (24 weeks) which subside as the advanced fibrosis develops by week 52.

#### Oncogenic signaling increases with disease progression

**Figure 6:**
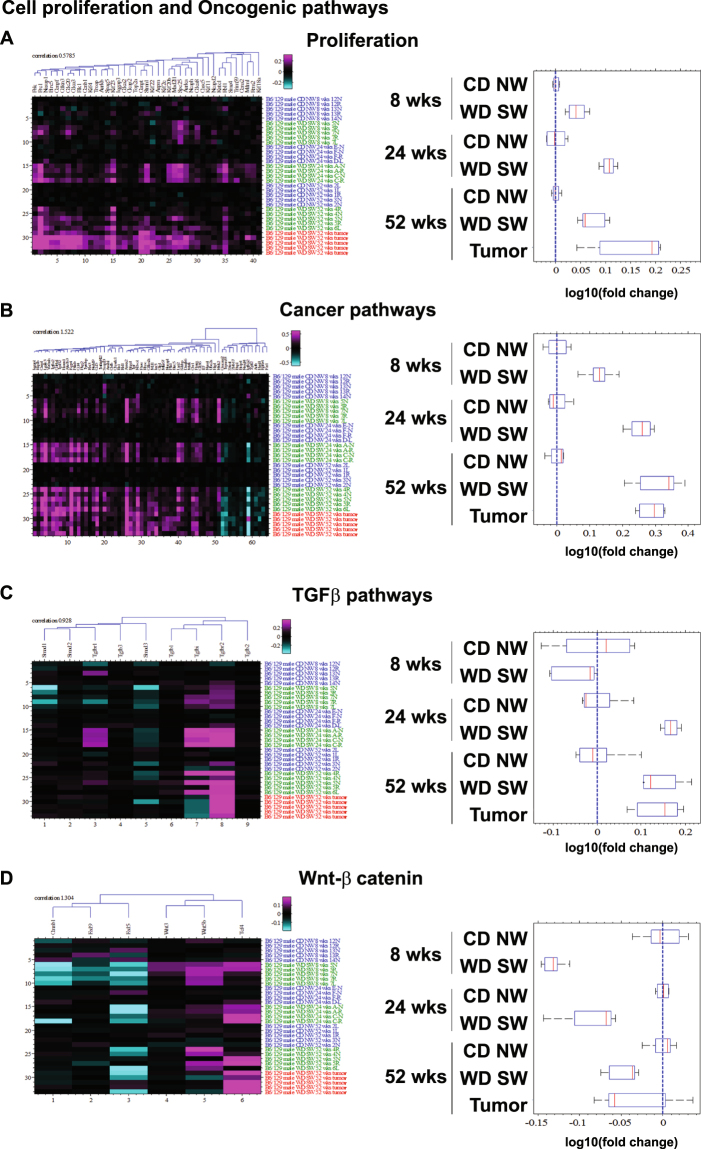
Changes in cell proliferation and oncogenic pathways. Heat maps resulting from hierarchical clustering and box plots with averages for genes implicated in (**A**) Proliferation (41 genes averaged), (**B**) Cancer pathways (51 genes averaged), (**C**) TGFβ signaling (2 genes averaged), (**D**) Wnt-β catenin (3 genes averaged) pathways with a fold-change greater than ±1.5 from WD SW liver samples as compared to CD NW at 8, 24 and 52 weeks or from liver tumors at 52 weeks as compared to WD SW 52 weeks with a false discovery rate (FDR) <0.1. Boxes show 25^th^ and 75^th^ percentile, whiskers show 5^th^ and 95^th^ percentile, red line is the median average score, and blue dash line shows no change compared with CD NW at 8, 24 and 52 weeks or compared to WD SW 52 weeks for liver tumors.

There was little evidence of activated oncogenic pathways in NAFL at week 8. However, cell proliferation (Fig. 6A) and oncogenic pathways (Fig. 6B–D) were significantly increased with development of steatohepatitis at 24 weeks. WD SW-mice had increased gene expression of *kinesin-like protein Kif20b* (Fig. 6A), an oncogene that can inhibit apoptosis and promote carcinogenic progression^23^, and Annexin A2 (Anxa2) (Fig. 6B) required for angiogenesis and metalloprotease activation, which are known to promote cancer cell migration^24^. The top up-regulated genes included *glycoprotein non-metastatic b* (*Gpnmb*) and *Galectin-3* (*Lgals3*) (Supplementary Table S5), both of which have been implicated in several cancers when overexpressed^25,26^.

With fibrosis progression and continued disease activity at week 52, the transcriptomic signature indicated a prominent activation of cell proliferation (Fig. 6A) and oncogenic pathways with activation of Wnt5b (Fig. 6B–D). *Protein regulator of cytokinesis 1* (*Prc1*), which has been associated with HCC recurrence and poor patient outcomes outcome^27^, was upregulated (Fig. 6A). Also, *Kif23*, which is induced by Prc1, was upregulated in WD SW-fed mice at 52 weeks (Fig. 6A) a result in agreement with a reported overexpression of *Kif23* in human HCC^28^. *Retinoblastoma-like protein 1* (*Rbl1*), another key gene involved in proliferation, was also upregulated (Fig. 6A). Other key oncogenic genes that were markedly upregulated included *Anxa2*, *Lgals3* and *transforming growth factor b receptor 2* (*Tgfbr2*) (Supplementary Table S5 and Fig. 6B). Thus, these results indicated activation of oncogenic pathways predominantly at advanced stages of the disease (52 weeks).

### Transcriptomic signature of NAFLD-associated HCC in DIAMOND mice

Ninety percent of DIAMOND mice at 52 weeks developed liver tumors^13^. When analyzing tumorous tissue as compared to adjacent non-malignant liver tissue, the 5 top predominant canonical pathways identified by IPA were related mostly to amino acid metabolism and included histidine degradation III (p = 8.58E-05, ratio 2/7), histidine degradation VI (p = 2.68E-04, ratio 2/12), glycine biosynthesis (p = 4.11E-03, ratio 1/2), biotin-carboxyl carrier protein assembly (p = 4.11E-03, ratio 1/2) and glutamine degradation (p = 4.11E-03, ratio 1/2) (Supplementary Table S2). Both *glutaminase 2* (*Gls2*) and histidine ammonia-lyase (*Hal*) were among the most downregulated genes (Supplementary Table S6), likely leading to glutamine and histidine accumulation which are all important nutrients to cell growth and proliferation^29^.

Most of the changes in gene expression relating to metabolic, cell injury, inflammation and fibrosis pathways were similar in the tumors to the adjacent tissue, but a noticeable increase in number of upregulated genes related to proliferation pathways was observed (Fig. 6A). Quantitative PCR analysis however indicated an additional increase in several inflammatory and fibrosis related genes in tumor tissue compared to adjacent non-malignant tissue (Supplementary Fig. S2 **)**. The overall direction of the changes was however similar. Among these upregulated genes, *baculoviral IAP repeat-containing protein 5* (*Birc5*), *Polo-like kinase 1* (*Plk1*), *cell division cycle 20 homolog* (*Cdc20*), *cell division cycle associated 3* (*Cdca3*) and cyclin B1 were all upregulated in liver cancers^30–33^. Also, *beclin 1* (*Becn1*), an autophagic gene associated with HCC^34^ and *MER proto-oncogene receptor tyrosine kinase* (*Mertk*) (which regulates cell survival, migration and differentiation and phagocytosis upon binding of ligands including Lgals3) were among the top up-regulated genes (Supplementary Table S5). Finally, IPA analysis identified the component of a transcriptional coactivator complex *mediator complex subunit 13* (*Med13*), the chaperone protein *prefoldin subunit 2* (*Pfdn2*) and the transcription factors *Hoxb8* and *E2f1* as top upstream regulators **(**Supplementary Table S3), indicative of cellular proliferation and activation of oncogenic pathways^35,36^.

### A composite dynamic molecular signature of NAFLD

At 8 weeks, the 5 top predominant canonical pathways identified include EIF2 signaling (p = 6.45E-11, ratio 59/148), acute protein response (p = 4.86E-08, ratio 55/156), unfolded protein response (p = 4.83E-07, ratio 25/53), mitochondrial dysfunction (p = 5.36E-07, ratio 48/138), and LXR/RXR activation (p = 9.3E-07, ratio 39/105) (Supplementary Table S2). Gene networks associated with lipid metabolism, molecular transport, and amino acid metabolism, protein synthesis and tissue differentiation were among the top-regulated network (Supplementary Table S4); and additional analysis of canonical pathways at 8 weeks revealed a significant increase in sterol regulatory element-binding transcription factor (Srebp)1c-mediated *de novo* lipogenesis (Fig. 1A) and β-oxidation/mitochondrial/peroxisomal fatty acid oxidation (Figs 1B, 2B).

**Figure 7:**
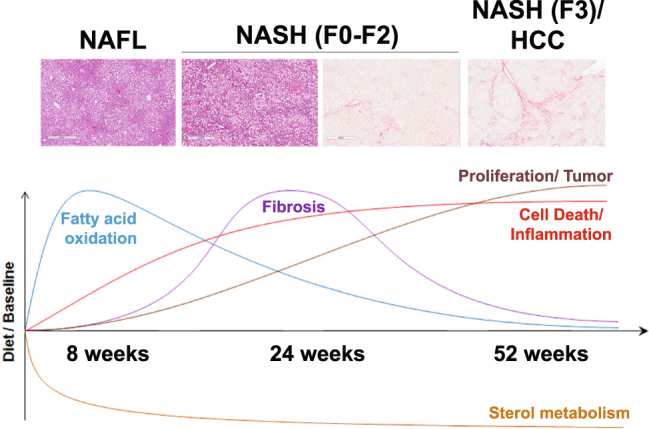
The molecular progression of NAFLD. First, a metabolic perturbation which triggers subsequent cell stress and inflammation driving cell death and turnover. Over time, inflammation and fibrogenic pathways become dominant while in advanced disease an inflammatory-oncogenic profile dominates. Upper part of the figure: Representative images of liver histology from DIAMOND mice at 8 (NAFL), 24 (Stage 0–2 fibrosis) and 52 weeks (Stage 3 fibrosis with HCC) (H&E or Sirius Red; original magnification, ×10).

With development of steatohepatitis (week 24), these changes persisted but started to trend down while inflammatory, fibrogenic and oncogenic signaling pathway gene expression increased (Supplementary Table S2 **)**. The top gene networks shifted to reflect the increase in inflammation and fibrosis (Supplementary Table S4). Disease progression (week 52) was associated with persistent cell stress and inflammation and increasing fibrogenic and oncogenic signaling. The activation of lipid metabolic, cell stress, inflammatory, fibrogenic and oncogenic signaling with various phenotypes and at varying time points while continuing on the same diet is summarized and shown in Fig. 7.

## Discussion

There is substantial heterogeneity with respect to histological features, risk factor profile and rates of disease progression in those with NAFLD^37^. While many molecular mechanisms attributable to disease development and progression have been described, it was not previously known when, in the course of the disease, these pathways get turned on or off. In the current study, using an extensively validated mouse model of progressive NAFLD, we demonstrate that the expression of genes over the course of the disease does not just follow a simple up- or down-regulation but that specific genes are differentially regulated at different points in the course of disease development and progression.

A key finding is that lipid metabolic pathway perturbations develop early along with features of cell stress and injury following initiation of diet-induced obesity. These data are in line with human data that indicate rapid development of hepatic steatosis and abnormal liver enzymes following initiation of an obesogenic diet^38^. Increased *SREBP-1c* mRNA and its downstream targets involved in *de novo* lipogenesis were increased early but decreased with disease progression despite continued intake of the diet along with insulin resistance. Along with increased lipid synthesis, there is also simultaneous activation of lipid oxidation pathways particularly PPAR-α in mice with NAFL which declines over time. This time-dependent change in PPARα activation provide a potential explanation for discrepant reports of both increased and decreased fatty acid β oxidation in “snap-shot” studies of humans with NAFLD^39,40^.

It is interesting to also note that the *Pnpla3* gene expression increased early and remained turned on throughout the course of the disease. This is likely to reflect an attempt by the liver to mobilize accumulated triglycerides and would be expected to further enhance the impact of loss of function mutations in this gene. Another pathway that appears to be important throughout the course of the disease is the FXR signaling pathway which is activated early and remains activated even at 52 weeks when bridging fibrosis is present. This was associated with increased expression of *Cyp7a1*, the rate limiting enzyme for bile acid synthesis from cholesterol early in the course of the disease. These further validate FXR as a relevant target in the development of the disease and thus for treatment of the disease.

Another key aspect of progressive NAFLD is hepatocyte apoptosis^41^. Altered mitochondrial function, oxidative stress, ER stress, autophagy and lysosomal dysfunction have all been implicated as drivers of apoptosis in NASH in humans^39,42–44^. The current study demonstrates that these pathways are activated in the DIAMOND mice. Furthermore, both drivers of the intrinsic *e.g*. oxidative stress and nrf2 and extrinsic e*.g*. Fas pathways are activated early in the course of the disease and remain increased with disease progression suggesting that these may be relevant therapeutic targets along the course of the disease. *Keap1* was also decreased in our model resulting in hepatocyte toxicity^45^. In accord with our previously published data^13^, ER stress-induced *Chop* was also upregulated in the later stages of our model, and likely contributed to liver injury^46^. In hepatocyte, both of these pathways result in mitochondrial permeabilization thereby activating effector caspases and PARP cleavage. *Vdac3*, which controls mitochondrial permeability, *caspase 6* and *7*, and *Parp1* were all upregulated in our model.

The principal difference in the transcriptome with disease progression from NAFL to NASH was the activation of inflammatory and fibrogenic signaling pathways. The transcriptome signature of WD SW-fed mice was dominated by activation of inflammation-related pathways with increased expression of genes related cytokines/chemokines, toll-like receptor and inflammasome, all of which have been described in human NAFLD^47^. Particularly, a robust increase in INFγ was observed in our model and could result from the accumulation of NK and NKT cells, induced by high fat diet administration as reported previously^48^, with subsequent activation of macrophages or KC. In addition, the transcriptomic signature of 24 weeks-mice fed a WD SW diet was indicative of the recruitment of eosinophils and dendritic cells to sites of inflammation, which have been associated with NAFLD progression^20^.

Not unexpectedly, fibrosis progression was associated with increased activation of fibrosis-associated pathways. The data indicate that multiple potential mechanisms from increased fibrogenic pathway activation e.g. TGF-β, increased fibrolytic pathway activation of several metalloproteinases, tissue inhibitor of metalloproteinase-1 (TIMP-1), Hedgehog signaling (increased Gli3) and connective tissue growth factor activation mediate fibrosis progression in this model and are concordant with previous reports of their activation status in humans with NASH^49^.

By the time advanced disease with HCC was present, the transcriptome was dominated by activation of several cell proliferation and oncogenic pathways. Diet-induced HCCs are known to be accompanied by differential expression of genes involved in the Myc, NFκB and TGFβ networks^12^. In our model, activation of cancer-related pathways were related to increased expression of *Anxa2* important for angiogenesis^24^, *Lgals3* overexpressed in lung cancers^26^ and *tumor growth factor b receptor 2* (*Tgfbr2*) and *Wnt5b*, likely indicative of an increased TGFβ and Wnt signaling.

Another attractive target to emerge from this study is the microRNA miR34a. We have previously shown this to be increased in NASH^50^ and this is also increased early in the DIAMOND model. Overexpression of miR34a has been linked to the severity of NASH and also development of HCC^51^.

Together the results of this study allow development of a time-dependent dynamic transcriptomic model of progressive NAFLD that relates specific pathway activation status to both time course following initiation of a WD SW diet and also the liver histology. They further demonstrate that despite similar histological activity with early stage disease versus late stage disease, there are numerous differences in the status of cell stress and inflammatory pathway activation. These may provide insights in to the variable response to therapeutic interventions in the future.

It is of course important to note that “mice” are not “men” and that animal models only approximate human disease and do not replicate the human condition in its entirely. True cirrhosis, as seen in humans, is not seen in the DIAMOND model which develops advanced bridging fibrosis. Also, it has a higher incidence of HCC than is seen in humans. Another key difference is that cholesterol synthesis is suppressed in this model whereas it is increased in humans with NASH^52^. Thus, it will be important to ultimately confirm the findings of this model in longitudinal cohorts of humans with multiple liver biopsies.

Regardless, the model demonstrates a high level of concordance with pathways known to be important in humans and will hopefully serve to test specific hypotheses related to targeting therapies based on transcriptome. We further anticipate and hope that this will provide a useful tool in development of Precision Medicine approaches to the management of NASH.

## Methods

### Animals and Diets

The initial physiological, metabolic and histologic characterization of our unique, isogenic mouse strain derived from a C57BL/6J and 129S1/SvImJ background (B6/129) has been previously published^13^. Male mice (8–12 weeks of age) were fed *ad lib* a high fat diet, high carbohydrate diet (Western Diet, WD) with 42% calories from fat and containing 0.1% cholesterol (Harlan TD.88137) with a high fructose-glucose solution (SW, 23.1g/L d-fructose +18.9 g/L d-glucose), as previously described^13^. Control mice were fed a standard chow diet (CD, Harlan TD.7012) with normal tap water (NW). All mice were housed in a 12 h light–12 h dark cycle in a 21–23 °C facility and were euthanized at varying time points following initiation of dietary intervention. All procedures were performed according to protocols approved by the Animal Care and Use Committee of Virginia Commonwealth University (IACUC AM 10154).

### RNA Isolation and Microarray Analysis of Liver Gene Expression

RNA was extracted from liver samples using a commercially available kit from QIAGEN (74104) following the manufacturer’s protocol. RNA quality was determined using the Agilent Bioanalyzer (Agilent) with RNA 6000 Nano Kits (Agilent, 5067–1511). Total RNA yield, 260/280, and 260/230 ratios were measured using a NanoDrop spectrophotometer (Thermo), and reported RNA integrity numbers (RIN) were >8.

Mouse whole-genome profiling was performed using the Illumina mouse WG6 Expression BeadChip kits (Illumina) (Cat #BD-201-0602) as described previously^13^. Raw intensity values were acquired using the HiScan microarray scanner and imported to GenomeStudio using the Gene Expression Module (both from Illumina). Raw data-sets without normalization or background correction on the arrays were exported from GenomeStudio (Illumina) to MATLAB (MathWorks), where they were log-transformed. We also applied quantile normalization to the raw expression intensities and removed batch differences using bridging samples.

### Statistical and Pathway Analysis

Differential gene expression between different dieting groups was determined using standard t-test for all matching time points (8, 24, and 52 weeks) separately. Multiple-testing correction was performed using Benjamini-Hochberg false discovery rate calculation from the obtained t-test p-value distributions. Differentially expressed genes were defined as those with FDR <0.1 and fold-change of at least 1.5 in any of the time points. Ingenuity Pathway Analysis (IPA, Qiagen) was used to perform biological pathway enrichment analysis on the differential gene expression data sets. Briefly, week 8, 24 and 52 gene expression data (fold-change, fdr and p-value for each probe ID) were uploaded to IPA and the IPA core analysis were performed with a false discovery rate cut off 0.1 for 8 and 24 weeks and 0.125 for 52 weeks. Top canonical pathways and upstream regulators for each of the time points were identified based on p-value, gene enrichment ratio and consistent directionality of the gene changes and were used for further interpretation and visualization as described below. For a detailed description of Ingenuity Pathways Analysis, visit www.Ingenuity.com.

The differentially expressed genes that belong to significantly enriched pathways were hierarchically clustered in MATLAB to identify concertedly regulated genes, which were then averaged to calculate the composite score for the pathway regulation.

### Statistical Analysis

Descriptive statistics were used to describe the distribution of laboratory and histological findings using Excel and Prizm version 5.0. All data are expressed as the mean ± S.E. of the mean. Inter-group comparisons were made using analysis of variance (ANOVA) with *post hoc* Bonferroni correction for multiple comparisons as appropriate for normally distributed variables. The statistical analysis plan for bioinformatics analyses are noted in the section above. A two-tailed *p* value of 0.05 was set to establish statistical significance.

## Electronic supplementary material

Supplementary information

Supplementary Excel Sheet S1

Supplementary Excel Sheet S2

Supplementary Excel Sheet S3

Supplementary Excel Sheet S4
