# Author Correction: The Transcriptomic Signature Of Disease Development And Progression Of Nonalcoholic Fatty Liver Disease

**DOI:** 10.1038/s41598-020-65506-y

**Published:** 2020-05-15

**Authors:** Sophie Cazanave, Alexei Podtelezhnikov, Kristian Jensen, Mulugeta Seneshaw, Divya P. Kumar, Hae-Ki Min, Prasanna K. Santhekadur, Bubu Banini, Adolfo Gabriele Mauro, Abdul M. Oseini, Robert Vincent, Keith Q. Tanis, Andrea L. Webber, Liangsu Wang, Pierre Bedossa, Faridoddin Mirshahi, Arun J. Sanyal

**Affiliations:** 1 Division of Gastroenterology, Hepatology and Nutrition, Department of Internal Medicine, Virginia Commonwealth University, Richmond, VA USA; 2 Merck Research Laboratories, Kenilworth, NJ USA; 3 VCU Pauley Heart Center, Virginia Commonwealth University, Richmond, VA USA; 4 Department of Pathology, Hospital Beaujon, University Paris-Diderot, Paris, France

Correction to: *Scientific Reports* 10.1038/s41598-017-17370-6, published online 08 December 2017

In this Article, the authors neglected to include some potential conflict of interest. The Competing Interest section should read:

**Competing interests**

Dr. Sanyal is the founder of the company Sanyal Biotechnology that performs pre-clinical drug-testing using the DIAMOND model. Virginia Commonwealth University has ownership interests in Sanyal Bio.

