# Supplementary material for: The Transcriptomic Signature Of Disease Development And Progression Of Nonalcoholic Fatty Liver Disease
Source: Sci Rep. 2017 Dec 8;7:17193. doi: 10.1038/s41598-017-17370-6 (PMC5722878; doi:10.1038/s41598-017-17370-6)

THE TRANSCRIPTOMIC SIGNATURE OF DISEASE DEVELOPMENT AND PROGRESSION OF NONALCOHOLIC FATTY LIVER DISEASE

Sophie Cazanave PhD[1*], Alexei Podtelezhnikov PhD[2], Kristian Jensen PhD[2], Mulugeta Seneshaw MSc[1], Divya P. Kumar PhD[1], Hae-Ki Min PhD[1], Prasanna K. Santhekadur PhD[1], Bubu Banini MD PhD[1], Adolfo Gabriele Mauro PhD[3], Abdul Oseini MD[1], Robert Vincent[1], Keith Q. Tanis PhD[2], Andrea L. Webber PhD[2], Liangsu Wang PhD[2], Pierre Bedossa MD[4], Faridoddin Mirshahi MSc[1] and Arun J. Sanyal MBBS, MD[1*].

Table of contents:

Supplementary Methods……………………………………………………………………….p2
Supplementary Fig. S1……………………………………………………………………………p3
Supplementary Fig. S2……………………………………………………………………………p4
Supplementary Fig. S3……………………………………………………………………………p5
Supplementary Fig. S4…………………………………………………………………………....p6
Supplementary Fig. S5…………………………………………………………………………....p7
Supplementary Fig. S6…………………………………………………………………………....p8
Supplementary Fig. S7…………………………………………………………………………....p9
Supplementary Figure Legends…………………………………………………………...p10
Supplementary Table S1……………………………………………………………………...p11
Supplementary Table S2…………………………………………………………………….…..p12
Supplementary Table S3……………………………………………………………………...p13
Supplementary Table S4…………………………………………………………………......p14
Supplementary Table S5……………………………………………………………………...p15
Supplementary Table S6……………………………………………………………………...p16
Supplementary Table S7……………………………………………………………………...p17

SUPPLEMENTARY METHODS

***Immunoblot Analysis*:** Whole cell lysates were prepared as previously described (*1*). Equal amounts of protein (20 to 50 µg) was resolved by SDS-PAGE on a 4%-12% NuPAGE Novex Bis-Tris Mini Gels (Invitrogen), then transferred to nitrocellulose membranes, and incubated with primary antibodies. Membranes were incubated with appropriate horseradish peroxidase-conjugated secondary antibodies (Cell Signaling Technology, Danvers, MA). Bound antibody was visualized using the SuperSignal chemiluminescent kit (Thermo Scientific Pierce Biotechnology, Inc, Rockford, IL) and the chemiluminescent signal was detected using Phenix blue X-Ray film (Phenix Research Product, Hayward, CA) or the FluorChem E chemiluminescence system (ProteinSimple, Santa Clara, CA).

Antibodies and Reagents: Antibodies used were obtained from the following sources: rabbit anti-JNK (#9252), rabbit anti-phospho-JNK (Thr183/Thr185) (#9251), rabbit anti-Fatty acid synthase (#3189), rabbit anti-Acetyl-CoA Carboxylase (#3662), rabbit anti-p42/p44 (p-Erk1/2) (4377), rabbit anti-phospho-p42/p44 (Erk1/2) (#4695) (Cell Signaling Technology). β-actin-HRP (ab49900) was purchased from Abcam.

Quantitative Real-Time Polymerase Chain Reaction: Total RNA was extracted using Trizol reagent and the purified RNA was reverse transcribed using high capacity reverse transcription kit (Applied Biosystems, Foster city, CA). Real time PCR was carried out using CFX96 Touch™ Real-Time PCR system (Biorad) as described previously (*2*). Cycle threshold (Ct) values were obtained and the relative fold change in gene expression was calculated as $2^{-\Delta\Delta Ct}$. The change in mRNA expression was calculated using differences of Ct values compared to housekeeping gene, Glyceraldehyde-3-phosphate dehydrogenase (GAPDH). Primer used are listed in **Supplementary Table S7**.

Immunohistochemistry: Formalin-fixed, paraffin-embedded liver tissue slides were used. Liver sections were deparaffinized and rehydrated. Following antigen retrieval, slides were incubated with primary antibody for ASC (1:250; Sigma-Aldrich) overnight at 4 °C. Anti-rabbit Alexa Fluor 594-

conjugated secondary antibody (1:100) was applied at room temperature for 2 h. To overcome for the auto fluorescence of the liver tissue, we used a solution of 1% Sudan black in 70% ethanol for 5 min. Counterstaining was accomplished with 4′,6-diamidino-2-phenylindole (DAPI) 1:20.000 for 5 min and the slides were coverslipped with SlowFade Antifade (Invitrogen). Negative controls with nonspecific IgG were run in parallel. Images were acquired with an Olympus IX70 microscope and CellSens software (Olympus life Science) using a 40× objective (400× magnification). Color composite images were ultimately generated.

1. A. Asgharpour *et al.*, A diet-induced animal model of non-alcoholic fatty liver disease and hepatocellular cancer. *J Hepatol* **65**, 579-588 (2016).
2. P. Puri *et al.*, Activation and dysregulation of the unfolded protein response in nonalcoholic fatty liver disease. *Gastroenterology* **134**, 568-576 (2008).

Supplementary Fig.S1

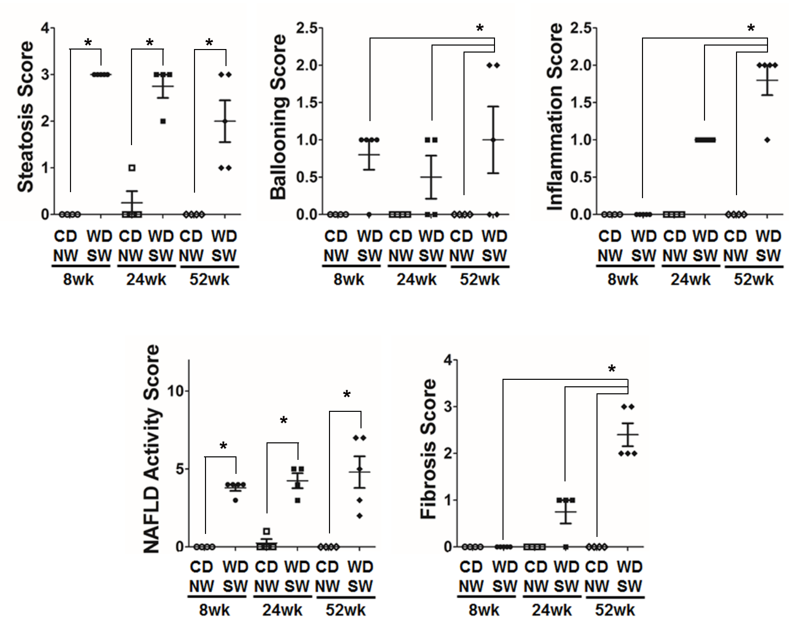

Supplementary Fig.S2

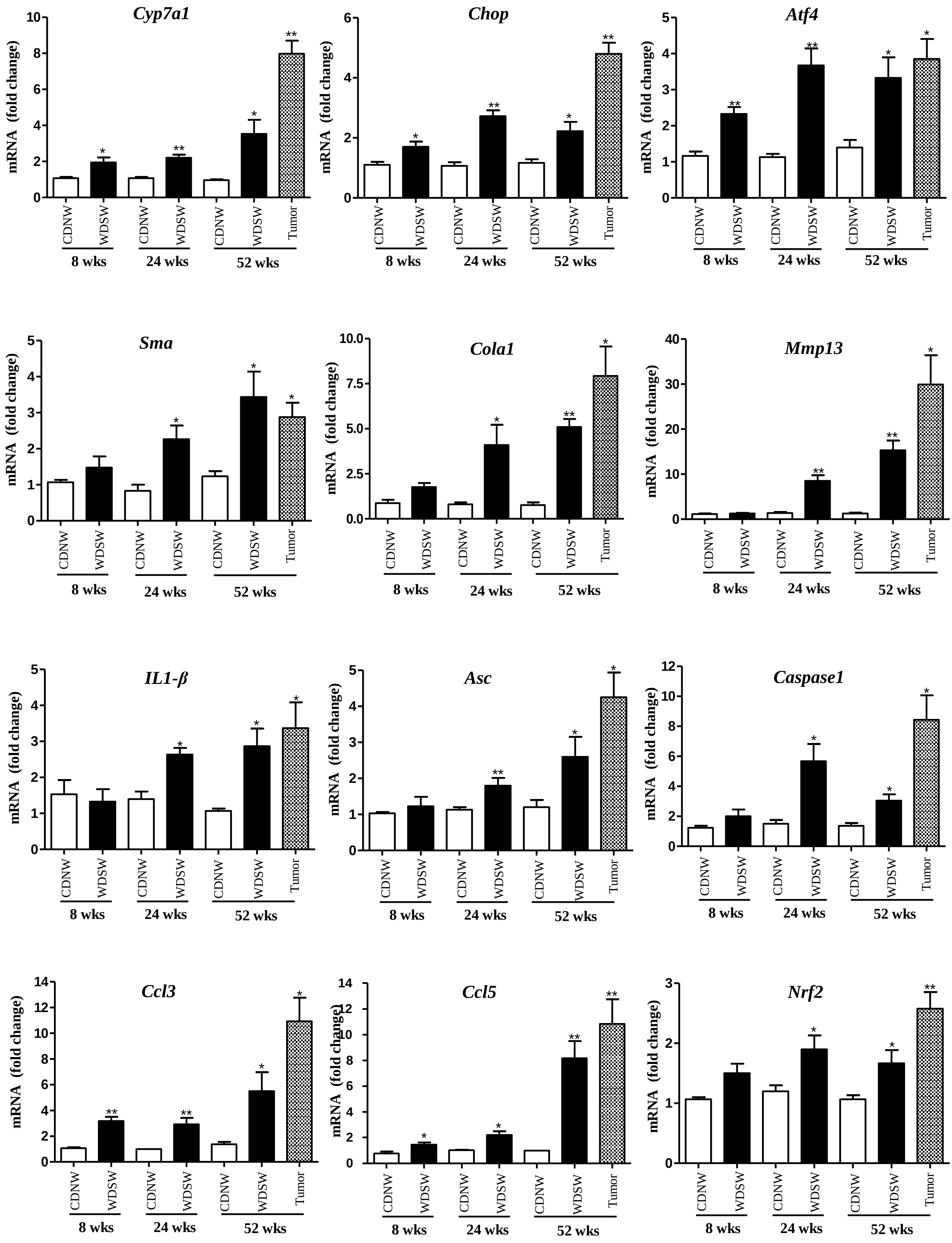

Supplementary Fig.S3

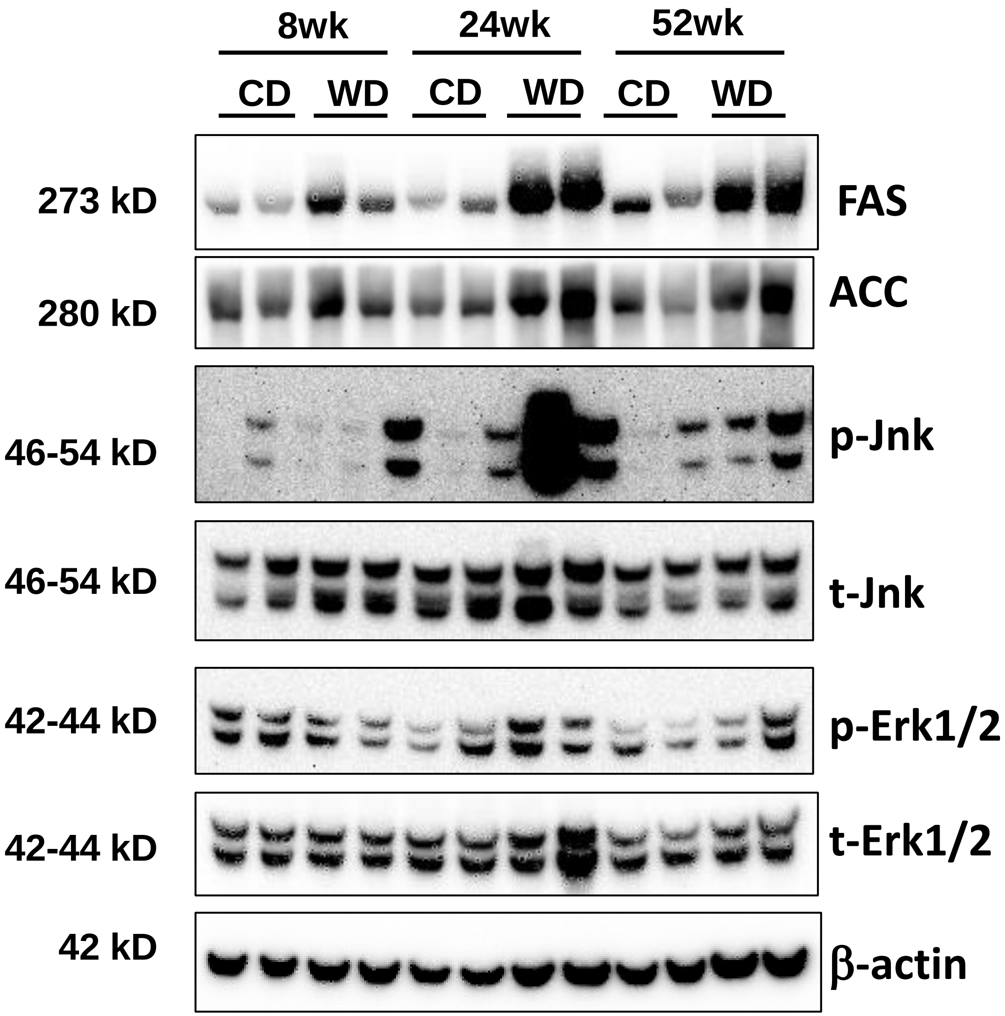

Supplementary Fig.S4: Chemokines/ Cytokines

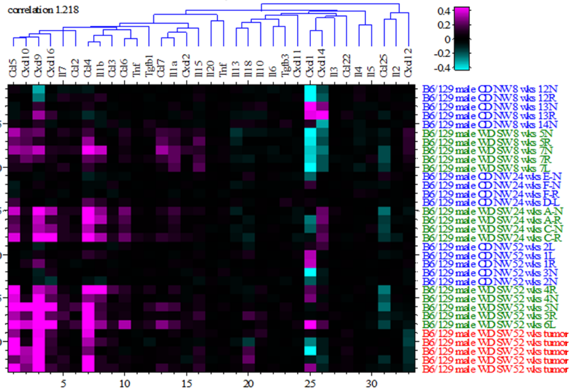

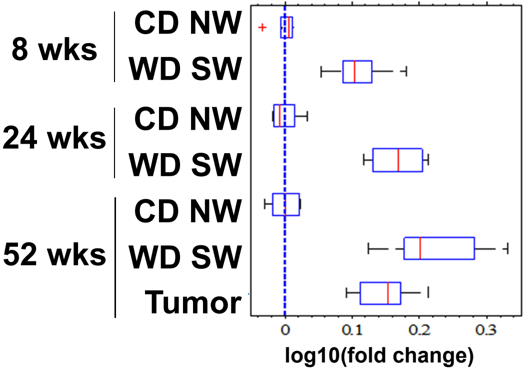

Supplementary Fig.S5: MAP Kinases

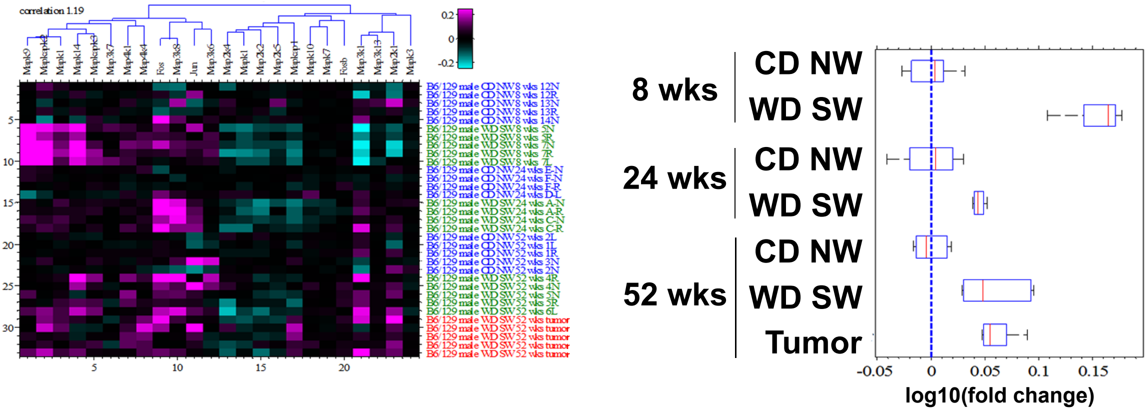

Supplementary Fig.S6: Hedgehog pathway

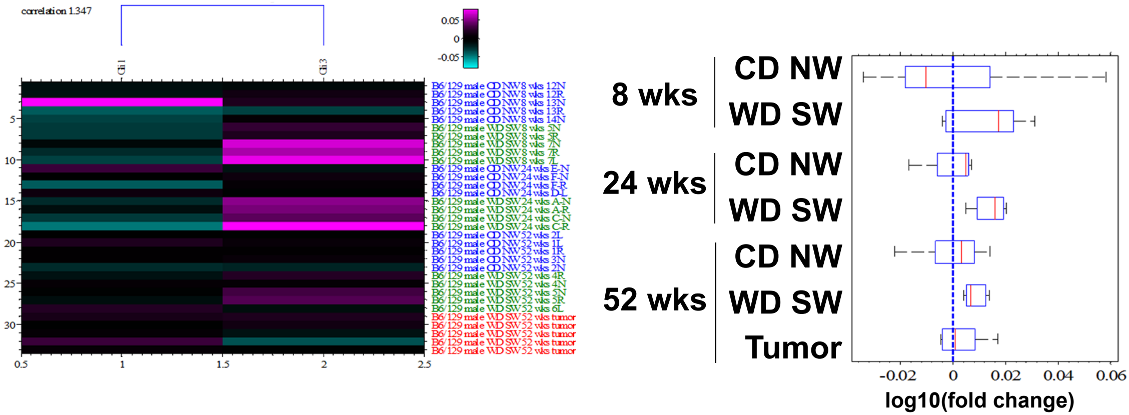

Supplementary Fig.S7

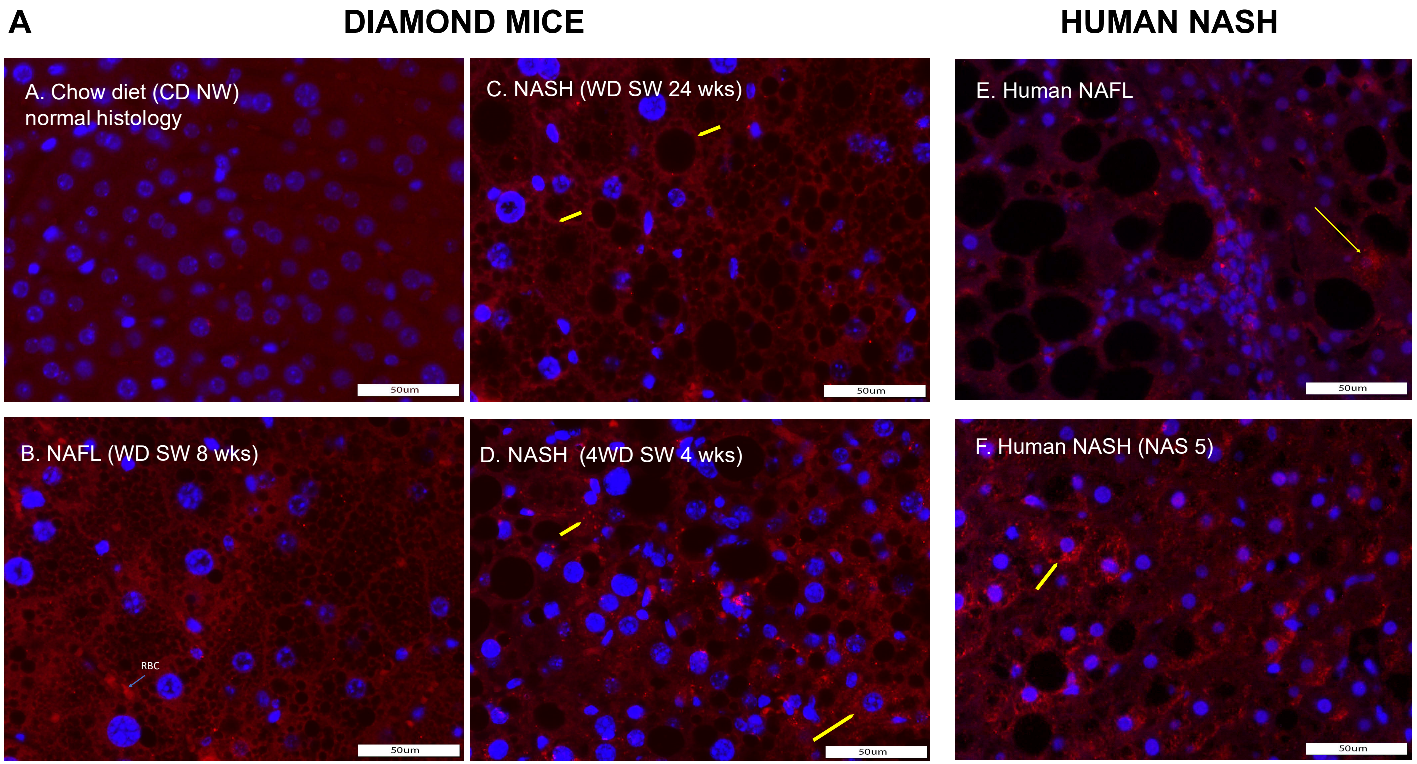

SUPPLEMENTARY FIGURE LEGENDS

Supplementary Fig.S1. Histology and fibrosis scores. B6/129 mice fed a chow diet (CD NW) or high fructose/glucose, high fat Western Diet (WD SW) for 8, 24 and 52 weeks. Histology score for steatosis, hepatocyte ballooning, lobular inflammation, NAFLD Activity Score and fibrosis were quantified from histology slides. Data are expressed as the mean ± SEM for 4-5 mice per group; *p <0.05.

Supplementary Fig.S2. Genes expression by real-time PCR. Total RNA was prepared from B6/129 mice fed a chow diet (CD NW) or high fructose/glucose, high fat Western Diet (WD SW) for 8, 24 and 52 weeks. *Cyp7a1, Chop, Atf4, Sma, Cola1, Mmp13, Il1-β, Asc, Caspase-1, Ccl3, Ccl5* and *Nrf2* mRNA expression were quantified by real-time PCR. Fold induction is relative to internal control GAPDH. Data represent mean ±S.E.M of n=4-5 mice; *p <0.05 or **p <0.001.

Supplementary Fig.S3. Changes in protein expression. Whole-cell lysates were prepared from B6/129 mice fed a chow diet (CD NW) or high fructose/glucose, high fat Western Diet (WD SW) for 8, 24 and 52 weeks. Immunoblot analysis were performed for FAS, ACC, phosphorylated Jnk (p-JNK), total Jnk (t-JNK), phosphorylated Erk1/2 (p-Erk1/2), total Erk1/2 and β-actin, a control for protein loading.

Supplementary Fig.S4. Changes in chemokines/cytokines. Heat maps resulting from hierarchical clustering and box plots with averages for genes implicated in chemokines/cytokines activation (16 genes averaged) with a fold-change greater than ±1.5 from WD SW liver samples as compared to CD NW at 8, 24 and 52 weeks or from liver tumors at 52 weeks as compared to WD SW 52 weeks with a false discovery rate (FDR) < 0.1. Boxes show 25th and 75th percentile, whiskers show 5th and 95th percentile, red line is the median average score, and blue dash line shows no change compared with CD NW at 8, 24 and 52 weeks or compared to WD SW 52 weeks for liver tumors.

Supplementary Fig.S5. Changes in MAPK pathways. Heat maps resulting from hierarchical clustering and box plots with averages for genes implicated in MAPK pathways (8 genes averaged) with a fold-change greater than ±1.5 from WD SW liver samples as compared to CD NW at 8, 24 and 52 weeks or from liver tumors at 52 weeks as compared to WD SW 52 weeks with a false discovery rate (FDR) < 0.1. Boxes show 25th and 75th percentile, whiskers show 5th and 95th percentile, red line is the median average score, and blue dash line shows no change compared with CD NW at 8, 24 and 52 weeks or compared to WD SW 52 weeks for liver tumors.

Supplementary Fig.S6. Changes in Hedgehog pathway. Heat maps resulting from hierarchical clustering and box plots with averages for genes implicated in Hedgehog pathway (2 genes averaged) with a fold-change greater than ±1.5 from WD SW liver samples as compared to CD NW at 8, 24 and 52 weeks or from liver tumors at 52 weeks as compared to WD SW 52 weeks

with a false discovery rate (FDR) < 0.1. Boxes show 25th and 75th percentile, whiskers show 5th and 95th percentile, red line is the median average score, and blue dash line shows no change compared with CD NW at 8, 24 and 52 weeks or compared to WD SW 52 weeks for liver tumors.

Supplementary Fig.S7. Activation of inflammasome in mouse and human liver tissue. Representative images of immunofluorescence staining for apoptosis speck-like protein containing a CARD domain ASC (red). Counterstaining with DAPI (blue). Yellow arrows indicate speck aggregation of ASC protein. Blue arrows indicate accumulation of red blood cells (RBC) in the tissue vessels. Liver biopsies were obtained from DIAMOND mice (A,B,C,D) or human NASH (E,F) (original magnification, ×40).

SUPPLEMENTARY TABLES

Supplementary Table S1. Serum biochemical and lipid profile.

**weeks of diet**	**8**		**24**		**52**	
**Type of diet**	**CD NW**	**WD SW**	**CD NW**	**WD SW**	**CD NW**	**WD SW**
**Weight**						
**- Body weight (g)**	**33.6** ± 2.8	**42.6** ± 1.1	**33.4** ± 3.2	**48.6** ± 1.4*	**30.1** ± 0.8	**44.0** ± 3.9**
**- Liver weight (g)**	**1.5** ± 0.2	**2.7** ± 0.2**	**1.2** ± 0.0	**3.5** ± 0.2	**1.1** ± 0.1	**4.8** ± 1.7*
**Biochemical parameters**						
**- AST (U/L)**	**136.8** ± 35.1	**345.6** ± 49.6*	**309.0** ± 86.6	**660.0** ± 217.0	**169.6** ± 17.0	**316.0** ± 81.5
**- ALT (U/L)**	**104.0** ± 22.9	**527.2** ± 157.4**	**275.0** ± 83.7	**605.0** ± 124.6	**128.0** ± 33.2	**361.6** ± 104.3
**- Alkaline Phosphatase (U/L)**	**59.2** ± 2.9	**94.4** ± 14.9*	**41.0** ± 1.0	**181.0** ± 22.1***	**108.8** ± 19.8	**167.2** ± 44.6
**- Cholesterol (mg/dL)**	**120.0** ± 12.5	**387.2** ± 49.5***	**84.0** ± 18.0	**375.0** ± 24.3***	**130.4** ± 6.6	**292.0** ± 50.2*
**- LDL-c (mg/dL)**	**45.6** ± 7.4	**218.4** ± 36.1***	**45.0** ± 9.8	**240.0** ± 12.0***	**51.2** ± 11.5	**189.6** ± 40.6**
**-HDL (mg/dL)**	**51.2** ± 6.4	**132.8** ± 17.3***	**28.0** ± 7.5	**117.0** ± 10.6	**53.6** ± 4.1	**84.0** ± 10.4***
**- Triglycerides (mg/dL)**	**118.4** ± 14.2	**180.8** ± 25.9	**48.0** ± 8.0	**92.0** ± 20.8	**87.2** ± 10.1	**92.0** ± 16.2

C57Bl/6J/129 S1/SvImJ (B6/129) mice were fed for 8, 24 or 52 weeks either a chow diet (CD NW) or a high fructose/sucrose, high fat Western diet (WD SW). Values are mean ± SEM for 4-5 mice per group; ****P < 0.001*, ***P < 0.01*, **P < 0.05* WD SW compared to CD NW. ALT, alanine aminotransferase; AST, aspartate aminotransferase; LDL-c, low-density lipoprotein-cholesterol.

Supplementary Table S2: Top

8 weeks			24 weeks			52 weeks			Liver Tumor		
**name**	**p-value**	**overlap**	**name**	**p-value**	**overlap**	**name**	**p-value**	**overlap**	**name**	**p-value**	**overlap**
**Eif2 Signaling**	6.45E-11	39.9 % (59/148)	**Intrinsic Prothrombin Activation Pathway**	5.30E-07	44.4 % (12/27)	**Fc Receptor-mediated Phagocytosis in Macrophages and Monocytes**	4.30E-06	12.6 % (11/87)	**Histidine Degradation III**	8.58E-05	28.6 % (2/7)
**Acute Phase Response Signaling**	4.86E-08	35.3 % (55/156)	**Fc Receptor-mediated Phagocytosis in Macrophages and Monocytes**	2.17E-05	23.0 % (20/87)	**Histidine Degradation III**	3.80E-04	42.9 % (3/7)	**Histidine Degradation VI**	2.68E-04	16.7 % (2/12)
**Unfolded Protein Response**	4.83E-07	47.2 % (25/53)	**Granulocyte Adhesion and Diapedesis**	2.49E-05	19.0 % (28/147)	**Actin Nucleation by Arp-WASp complex**	1.00E-03	11.8 % (6/51)	**Glycine Biosynthesis III**	4.11E-3	50.0 % (1/2)
**Mitochondrial Dysfunction**	5.36E-07	34.8 % (48/138)	**Hepatic fibrosis/ Hepatic Stellate Cell Activation**	2.93E-05	18.0 % (31/172)	**Epithelial Adherens Junction Signaling**	1.04E-03	7.4 % (10/135)	**Biotin-carboxyl Carrier Protein Assembly**	4.11E-3	50.0 % (1/2)
**LXR/RXR Activation**	9.30E-07	37.1 % (39/105)	**LPS/Il-1-mediated Inhibition of RXR Function**	7.49E-05	16.8 % (33/197)	**Production of Nitric Oxide and Reactive Oxygen Species in Macrophages**	1.47E-03	6.6 % (11/166)	**Glutamine Degradation**	4.11E-3	50.0 % (1/2)

Eif2, eukaryotic translation initiation factor 2; **LXR**, liver X receptor; **FXR**, farnesoid X receptor; **LPS**, lipopolysaccharide; **Il1**, interleukin 1; **Arp-WASp**, actin-related protein-Wiskott-Aldrich Syndrome

Supplementary Table S3: Top Upstream Regulators

8 weeks			24 weeks			52 weeks			Liver Tumor		
**name**	**p-value**	**predicted**	**name**	**p-value**	**predicted**	**name**	**p-value**	**predicted**	**name**	**p-value**	**predicted**
Pparα	6.33E-21	Activated	Tp53	2.02E-16		Gpd1	7.29E-07		Med13	7.72E-04	
Acox1	4.69E-18	Inhibited	Por	2.61E-16		Slc25a13	8.23E-07		Slc9a6	1.97E-03	
Rictor	1.21E-13		Infγ	5.35E-16	Activated	Infγ	2.95E-05	Activated	Hoxb8	1.97E-03	
Nfe2l2	5.61E-13	Activated	Acox1	8.27E-14	Inhibited	Dysf	3.06E-05		Pfdn2	1.97E-03	
Tp53	2.59E-10		Pparα	1.36E-13	Activated	miR-34a-5p and other miRNAs with seed GGCAGUG	4.32E-05		E2f1	3.40E-03	

Pparα, peroxisome proliferator-activated receptor α; **Acox1**, peroxisomal acyl-coenzyme A oxidase 1; **Rictor**, rapamycin-insensitive companion of mTOR; **Nfe2l2**, nuclear factor erythroid 2-related factor 2; **Tp53**, tumor protein p53; **Por**, cytochrome P450 oxidoreductase; **Infγ**, interferonγ; **Gpd1**, glycerol-3-phosphate dehydrogenase 1; **Slc25a13**, solute carrier family 25 member 13; **Med13**, mediator complex subunit 13; **Slc9a6**, solute carrier family 9 member 6; **Hoxb8**, homeobox b8; **Pfdn2**, prefoldin Subunit 2; **E2f1**, E2F transcription factor 1

Supplementary Table S4: Top Networks

8 weeks		24 weeks		52 weeks		Liver Tumor	
**Associated network functions**	**Score**	**Associated network functions**	**Score**	**Associated network functions**	**Score**	**Associated network functions**	**Score**
Lipid Metabolism, Molecular Transport, Small molecule Biochemistry	27	Lipid Metabolism, Small Molecule Biochemistry, Vitamin and Mineral Metabolism	32	Cancer, Organismal Injury and Abnormalities, Reproductive System Disease	40	Cellular Development, Cellular Growth and Proliferation, Organ Development	29
Amino Acid Metabolism, Small Molecule Biochemistry, Humoral Immune response	27	Cellular Assembly and Organization, Cell Morphology, Endocrine System Development and Function	32	Lipid Metabolism, Molecular Transport, Small molecule Biochemistry	35	Lipid Metabolism, Molecular Transport, Small molecule Biochemistry	19
Molecular Transport	27	Inflammatory Response, Tissue Morphology, Neurological Disease	30	Cellular Function and Maintenance, Cell Death and Survival, Hematological System Development and Function	31	Connective Tissue Development and Function, Connective Tissue Disorders, Developmental Disorder	2
Developmental Disorder, Hereditary Disorder, Metabolic Disease	25	Metabolic Disease, Cell Death and Survival, Organ Morphology	30	Cellular Movement, Cell Death and Survival, Hematological System Development and Function	29	Cell Death and Survival, Cellular Assembly and Organization, Cellular Function and Maintenance	2
Protein Synthesis, Connective Tissue Development and Function, Tissue Morphology	25	Lipid Metabolism, Small Molecule Biochemistry, Vitamin and Mineral Metabolism	28	Metabolic Disease, Endocrine System Disorders, Gastrointestinal Disease	24	Cellular Assembly and Organization, Connective Tissue Disorders, Hematological Disease	2

Supplementary Table S5: Top Up-regulated Molecules

8 weeks		24 weeks		52 weeks		Liver Tumor	
**Molecules**	**Exp. Value**	**Molecules**	**Exp. Value**	**Molecules**	**Exp. Value**	**Molecules**	**Exp. Value**
Rn18s	1.601	Gpnmb	1.268	Fcgr3a/Fcgr3b	1.049	Akr1b7	1.128
ApoA4	1.181	ApoA4	1.211	Ear2	0.880	Cib2	0.682
Cyp2b13/Cyp2b9	1.176	Mmp12	1.069	Lgals3	0.841	Mertk	0.252
Ca3	0.896	Lpl	0.944	C1qb	0.816	Becn1	0.231
Khk	0.895	Ear2	0.880	Anxa2	0.790	Atp6v0e2	0.225
Pnpla3	0.864	Cyp2b13/Cyp2b9	0.878	Cyth4	0.726	Rbms1	0.214
Hp	0.851	Lgals3	0.851	Cd52	0.725	Mylk	0.213
Ctse	0.849	Pnpla3	0.838	Mpeg1	0.724	Gprasp1	0.210
Adh1C	0.836	LOC102637129/S100a11	0.799	Sirpa	0.716	Agxt2	0.135
		Ear3	0.779	Cyp17a1	0.707	Pfdn5	0.126

Rn18s, 18S ribosomal RNA; **ApoA4**, Apolipoprotein A4; **Cyp2b13/Cyp2b9**, cytochrome P450, family 2, subfamily b, polypeptide 13/ cytochrome P450, family 2, subfamily b, polypeptide 9; **Ca3**, carbonic anhydrase III; **Khk**, ketohexokinase; **Pnpla3**, patatin-like phospholipase domain-containing protein 3; **Hp**, haptoglobin; **Ctse**, cathepsin E; **Adh1C**, alcohol dehydrogenase 1C; **Gpnmb**, glycoprotein non-metastatic b; **Mmp12**, metalloproteinase 12; **Lpl**, lipoprotein Lipase; **Ear2**, eosinophil-associated, ribonuclease A family, member 2; **Lgals3**, lectin, galactoside binding soluble 3; **LOC102637129/S100a11**, LOC102637129 protein S100-A11 pseudogene; **Ear3**, eosinophil-associated, ribonuclease A family, member 3; **Fcgr3a/Fcgr3b**, Fc fragment of IgG receptor 3a/ Fc fragment of IgG receptor 3b; **C1qb**, complement component 1, q subcomponent, b chain; **Anxa2**, annexin A2; **Cyth4**, cytohesin 4; **Cd52**, cluster of differentiation 52; **Mpeg1**, macrophage-expressed gene 1; **Sirpa**, signal-regulatory protein alpha; **Cyp17a1**, cytochrome P450, family 17, subfamily a, polypeptide 1; **Akr1b7**, aldo-keto reductase family 1, member b7; **Cib2**, calcium and integrin binding family member 2; **Mertk**, c-mer proto-oncogene tyrosine kinase; **Becn1**, beclin 1; **Atp6v0e2**, ATPase, H+ transporting, lysosomal V0 subunit E2; **Rbms1**, RNA binding motif, single stranded interacting protein 1; **Mylk**, myosin, light polypeptide kinase; **Gprasp1**, G protein-coupled receptor associated sorting protein 1; **Agxt2**, alanine-glyoxylate aminotransferase 2; **Pfdn5**, prefoldin subunit 5

Supplementary Table S6: Top Down-regulated Molecules

8 weeks		24 weeks		52 weeks		Liver Tumor	
**Molecules**	**Exp. Value**	**Molecules**	**Exp. Value**	**Molecules**	**Exp. Value**	**Molecules**	**Exp. Value**
Hsd3b4	-1.392	Hsd3b4	-1.465	Igfbp2	-0.586	Gls2	-1.192
Sqle	-1.128	Serpina1	-1.064	Serpine2	-0.507	Hal	-1.161
Serpina1	-1.093	Sqle	-1.036	Cyp2j9	-0.451	C9	-1.080
Tff3	-1.038	Asns	-0.899	Hal	-0.449	Serpina12	-0.910
Asns	-1.011	FoxQ1	-0.747	Agxt	-0.420	Uroc1	-0.682
Idi1	-0.907	Cyp7b1	-0.707	Raet1b	-0.415	Cadps2	-0.647
Egfr	-0.873	Idi1	-0.673	C5orf45	-0.388	Pigr	-0.571
Mup1	-0.854	Egfr	-0.622			Aldh1b1	-0.552
Selenbp1	-0.802	Cyp1a2	-0.598			Slco2a1	-0.470
C8b	-0.793	Susd4	-0.591			Ntrk2	-0.467

Hsd3b4, hydroxy-delta-5-steroid dehydrogenase, 3 beta- and steroid delta-isomerase 4; **Sqle**, squalene epoxidase; **Serpina1**, serpin family A member 1; **Tff3**, trefoil factor 3; **Asns**, asparagine synthetase; **Idi1**, isopentenyl-diphosphate delta isomerase; **Egfr**, epidermal growth factor receptor; **Mup1**, major urinary protein 1; **Selenbp1**, selenium binding protein 1; **C8b**, complement C8 beta chain; **FoxQ1**, forkhead box Q1; **Cyp7b1**, cytochrome P450, family 7, subfamily b, polypeptide 1; **Cyp1a2**, cytochrome P450, family 1, subfamily a, polypeptide 2; **Susd4**, sushi domain containing 4; **Igfbp2**, insulin-like growth factor binding protein 2; **Serpine2**, serpin family E member 2; **Cyp2j9**, cytochrome P450, family 2, subfamily j, polypeptide 9; **Hal**, histidine ammonia lyase; **Agxt**, alanine-glyoxylate aminotransferase; **Raet1b**, retinoic acid early transcript 1 beta; **C5orf45**, MRN complex interacting protein; **Gls2**, glutaminase 2; **C9**, complement component 9; **Serpina12**, serpin family A member 12; **Uroc1**, urocanase domain containing 1; **Cadps2**, calcium dependent secretion activator 2; **Pigr**, polymeric immunoglobulin receptor; **Aldh1b1**, aldehyde dehydrogenase 1 family, member B1; **Slco2a1**, solute carrier organic anion transporter family, member 2a1; **Ntrk2**, neurotrophic tyrosine kinase, receptor, type 2

Supplementary Table S7: Real-time PCR Primer sequences

Gene	Forward sequence (5'-3')	Reverse sequence (5'-3')	Product size (bp)
Cyp7a1	AGCAACTAAACAACCTGCCAGTACTA	GTCCGGATATTCAAGGATGCA	82
Chop	GCAGTCATGGCAGCTGAGTC	CGCAGGGTCAAGAGTAGTGA	180
Atf4	TCGATGCTCTGTTTCGAATG	AGAATGTAAAGGGGGCAACC	192
Sma	GTCCCAGACATCAGGGAGTAA	TCGGATACTTCAGCGTCAGGA	102
Cola1	GAAAACCCGAGGTATGCTTGA	GACCAGGAGGACCAGGAAGT	276
MMP13	TTCTGGTCTTCTGGCACACGCTTT	CCAAGCTCATGGGCAGCAACAATA	132
IL-1β	ACCTGTGTCTTTCCCGTGGACCT	TCATATGGGTCCGACAGCACGAG	198
Asc	CAGAGTACAGCCAGAACAGGACAC	GTGGTCTCTGCACGAACTGCCTG	146
Caspase-1	TCCGCGGTTGAATCCTTTTCAGA	ACCACAATTGCTGTGTGCGCA	84
Ccl3	ACTGCCTGCTGCTTCTCCTACA	AGGAAAATGACACCTGGCTGG	101
Ccl5	GCCCACGTCAAGGAGTATTTC	AACCCACTTCTTCTCTGGGTTG	111
Nrf2	GACATCCTTTGGAGGCAAGA	AGGCATCTTGTTTGGGAATG	280
Gapdh	AGAAACCTGCCAAGTATGATG	GGAGTTGCTGTTGAAGTCG	122

Supplemental Data 1: original Western-blots with surrounded cropped area

pERK

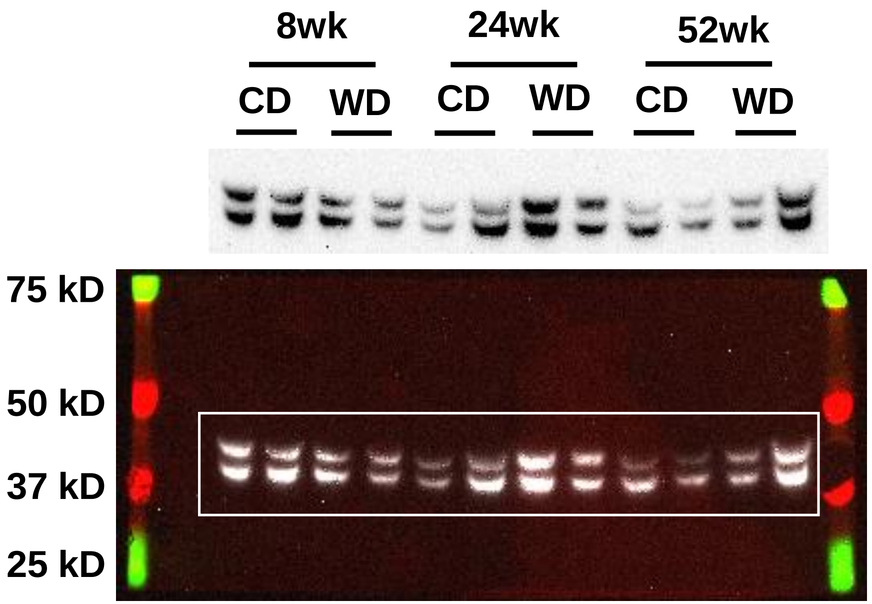

β-actin

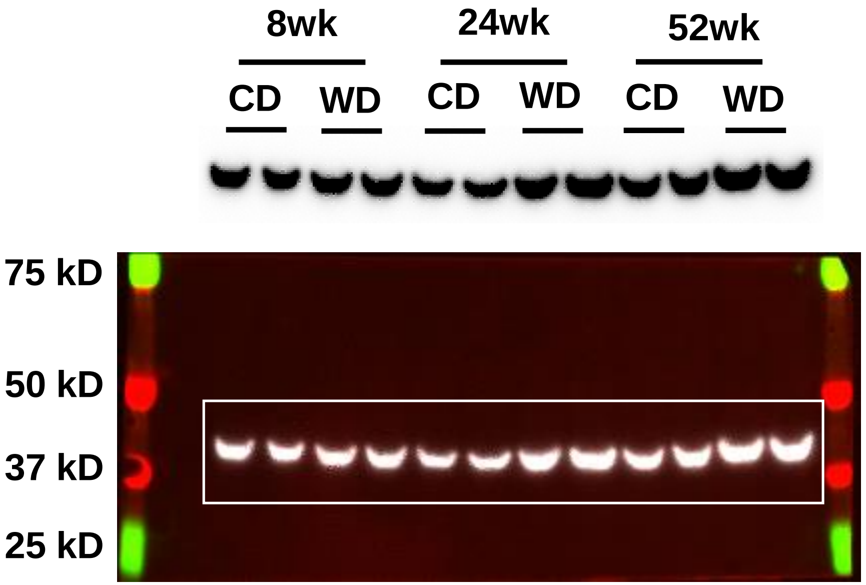

ERK

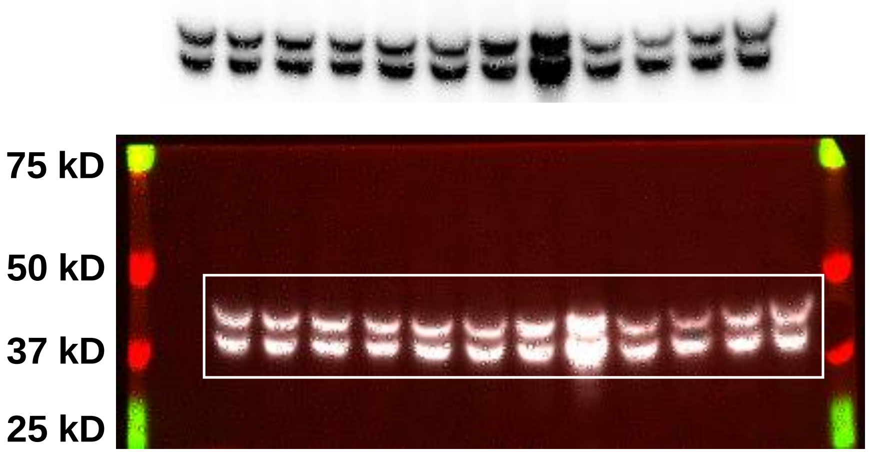

FAS

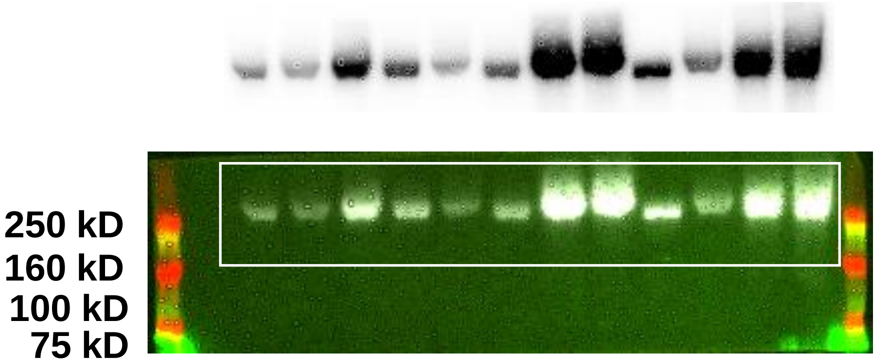

Supplemental Data 2: original Western-blots with surrounded cropped area

ACC

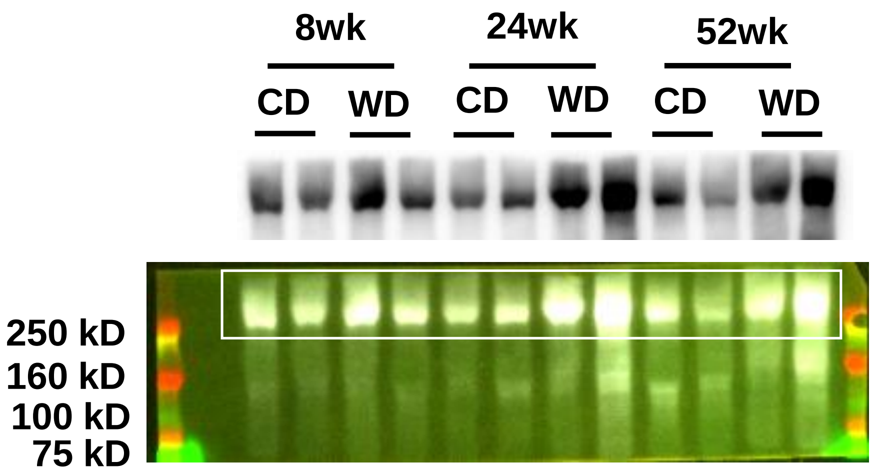

pJNK

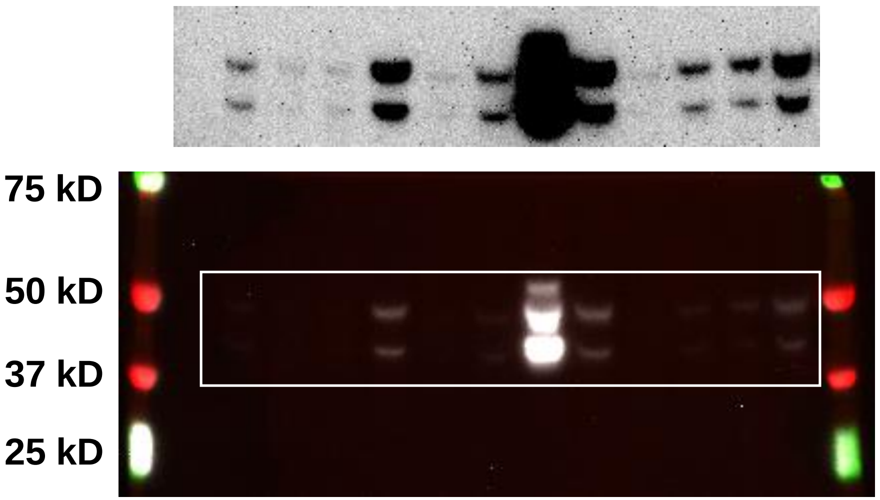

JNK

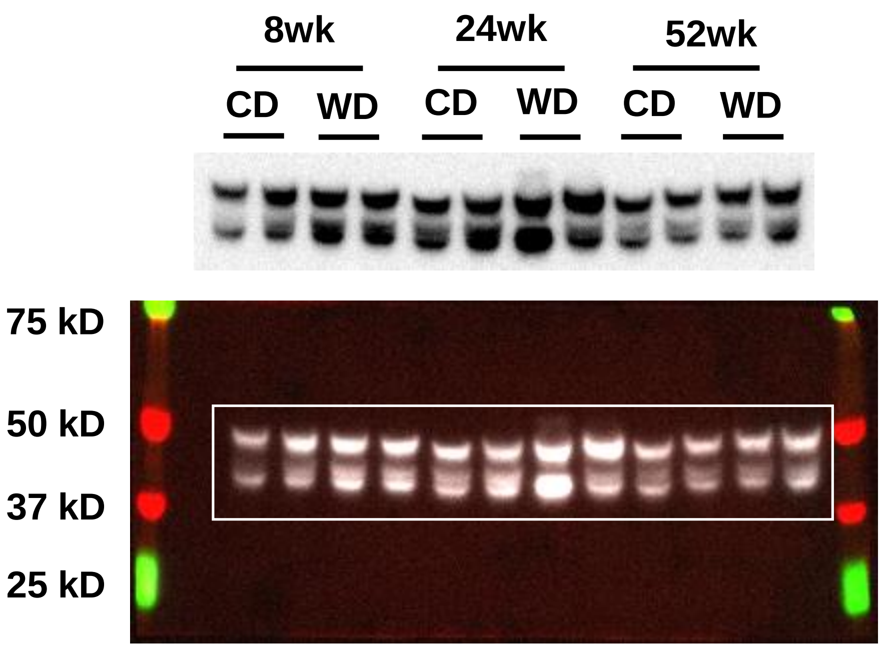

Supplement: Supplementary file 1 — Supplementary information [file 41598_2017_17370_MOESM1_ESM.pdf]
